# Supplementary figures and images for: Changes in the fine-scale genetic structure of Finland through the 20th century
Source: PLoS Genet. 2021 Mar 4;17(3):e1009347. doi: 10.1371/journal.pgen.1009347 (PMC7932171; doi:10.1371/journal.pgen.1009347)

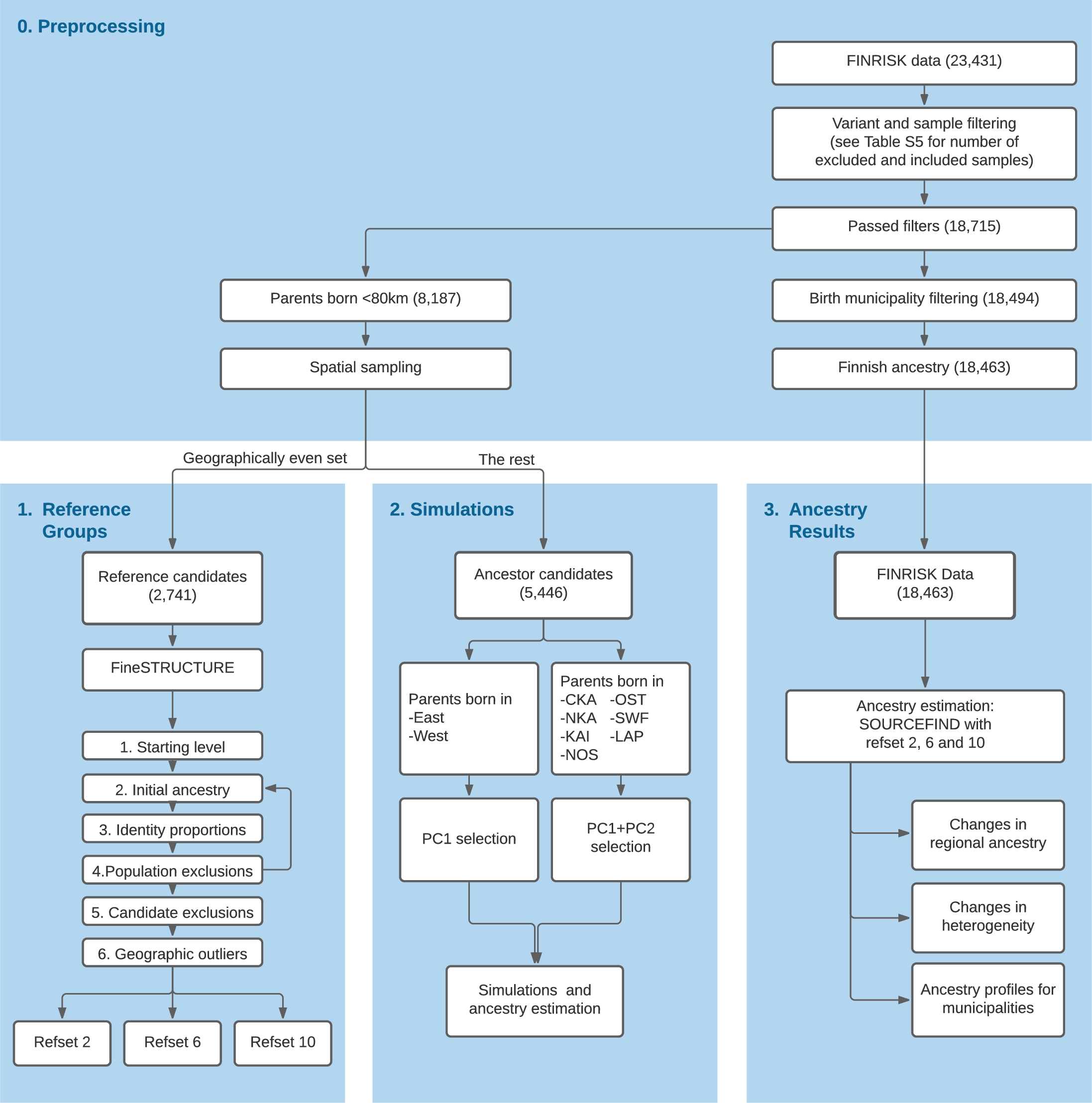

Supplement: S1 Fig — (TIF) [file pgen.1009347.s001.tif]

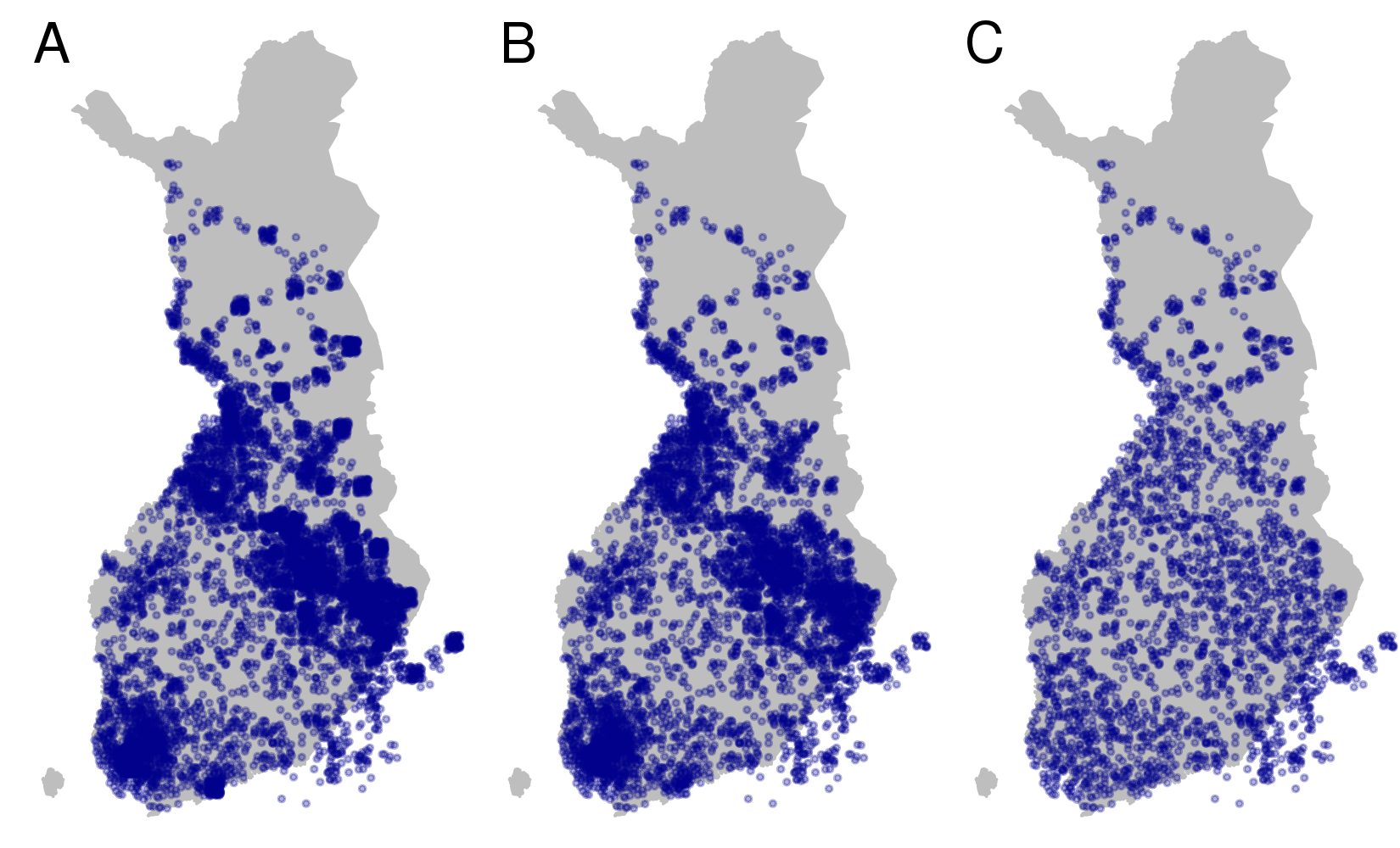

Supplement: S2 Fig — Geographic distribution of samples whose parents were born within 80 km from each other A) before the spatial sampling procedure, B) after excluding the individuals with a high number of local neighbors and C) after excluding the individuals with a high number of global neighbors. The individuals are displayed at the mean of their parents’ municipalities of birth after adding some jitter to the points to ensure the anonymity. Geographic outliers are excluded. (TIF) [file pgen.1009347.s002.tif]

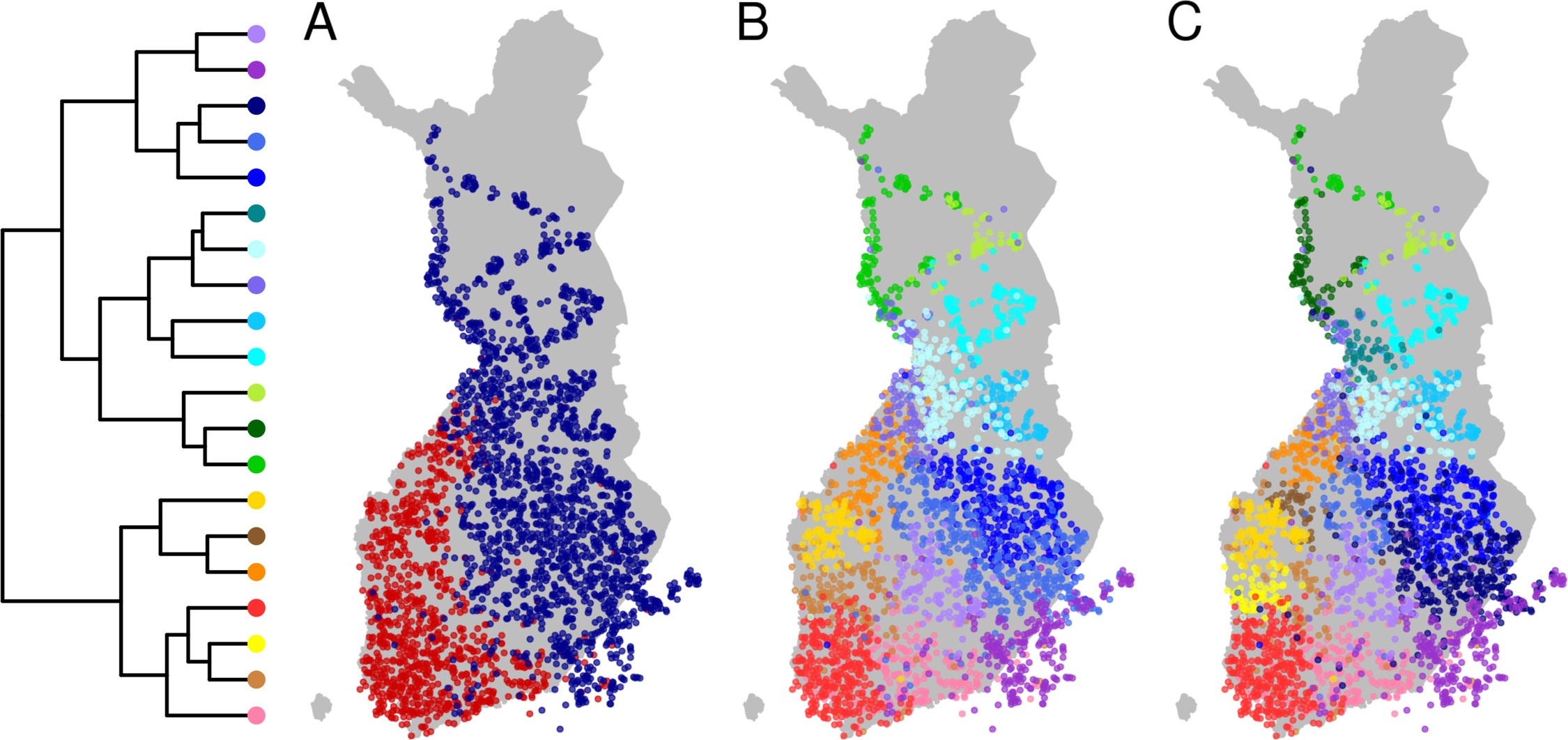

Supplement: S3 Fig — FineSTRUCTURE tree at level 20 and the corresponding populations on a map when the tree is cut at A) level 2, B) level 15 and C) level 20. (TIF) [file pgen.1009347.s003.tif]

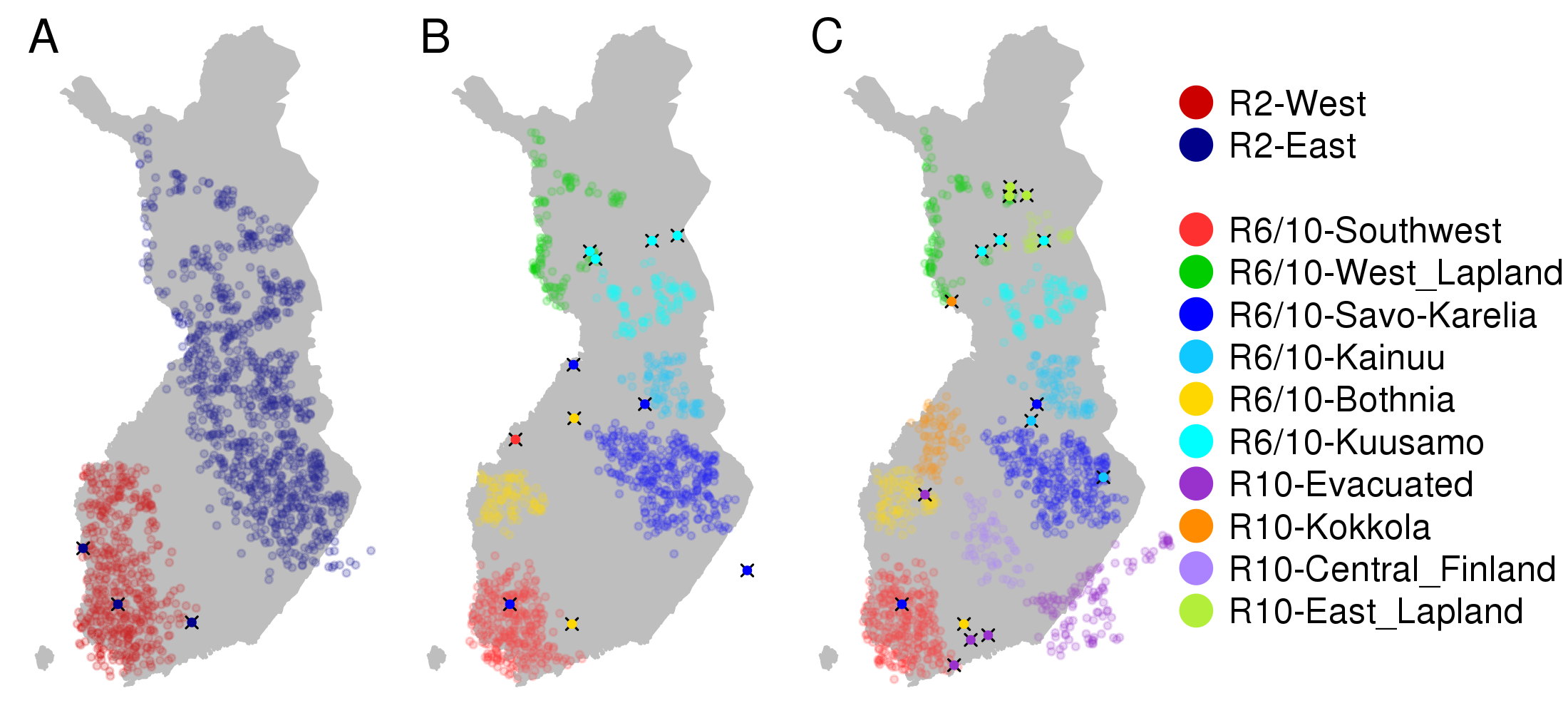

Supplement: S4 Fig — Maps shows the location of individuals excluded as geographic outliers of A) refset 2 (3 individuals), B) refset 6 (11 individuals) and C) refset 10 (16 individuals). The excluded outliers are highlighted with black X-marks. The included individuals are shown with pale colors. (TIF) [file pgen.1009347.s004.tif]

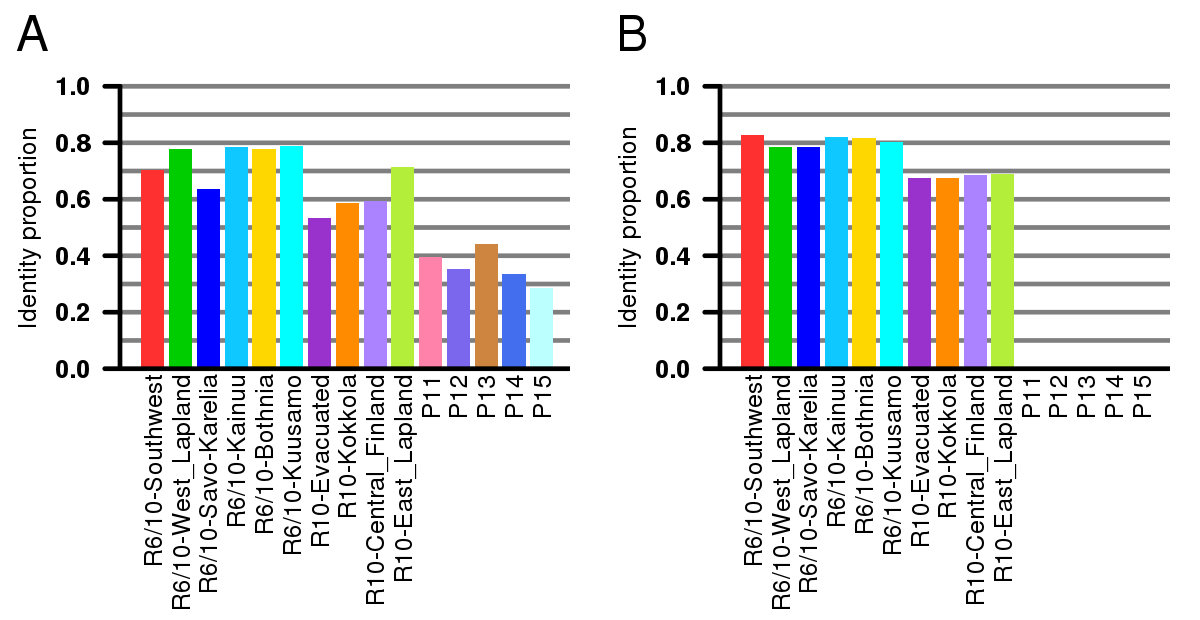

Supplement: S5 Fig — Panel A) shows the identity proportions when all 15 population were used as reference populations. Panel B) shows the identity proportions using only the 10 populations that show identity proportion above 0.50 in panel A. Colors correspond to the populations in S3B Fig. (TIF) [file pgen.1009347.s005.tif]

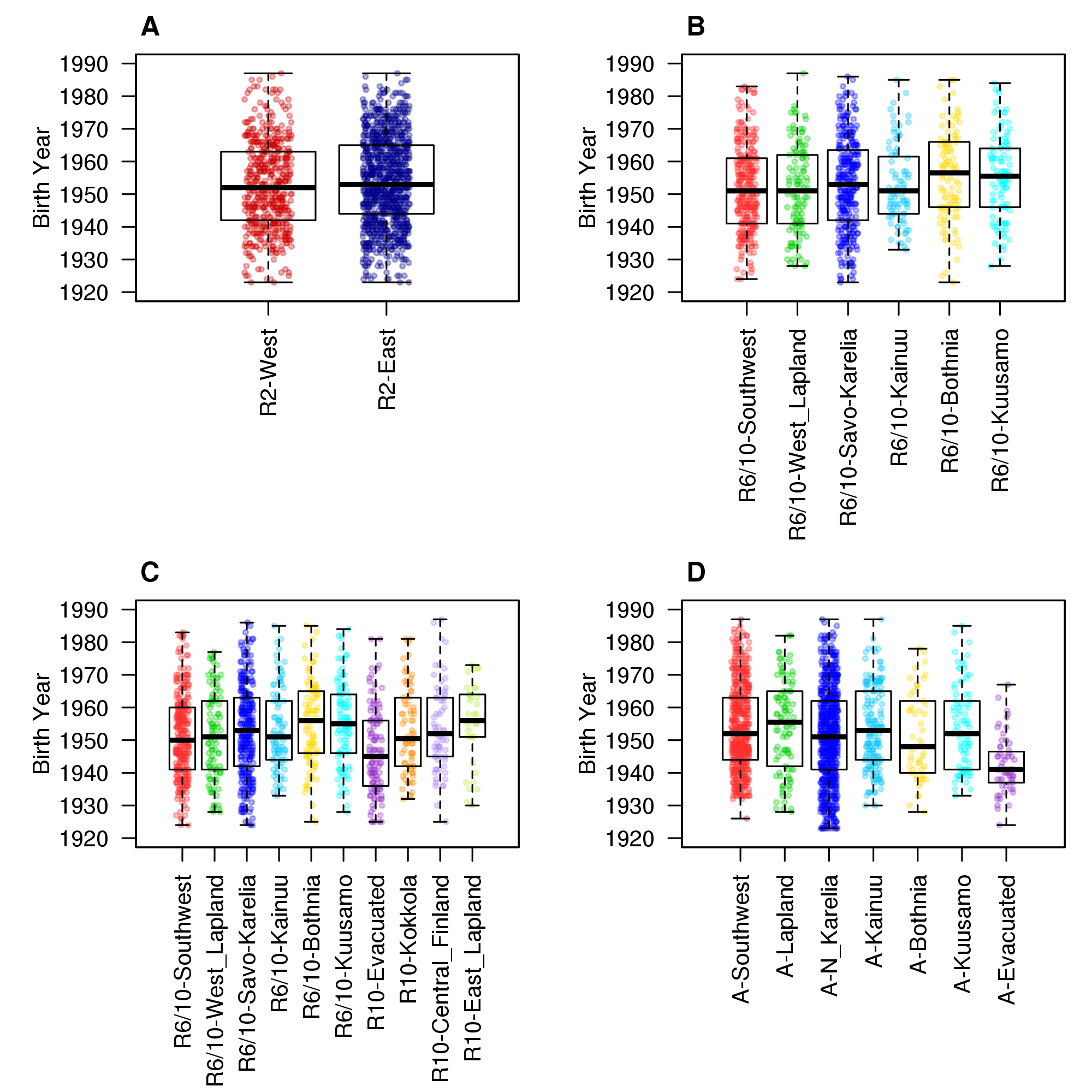

Supplement: S6 Fig — Age distributions of the reference groups of A) refset 2, B) refset 6, C) refset 10 and D) the ancestor candidates. The boxplot whiskers show the range, the boxes show the interquartile range and the dark line shows the median of the birth years. (TIF) [file pgen.1009347.s006.tif]

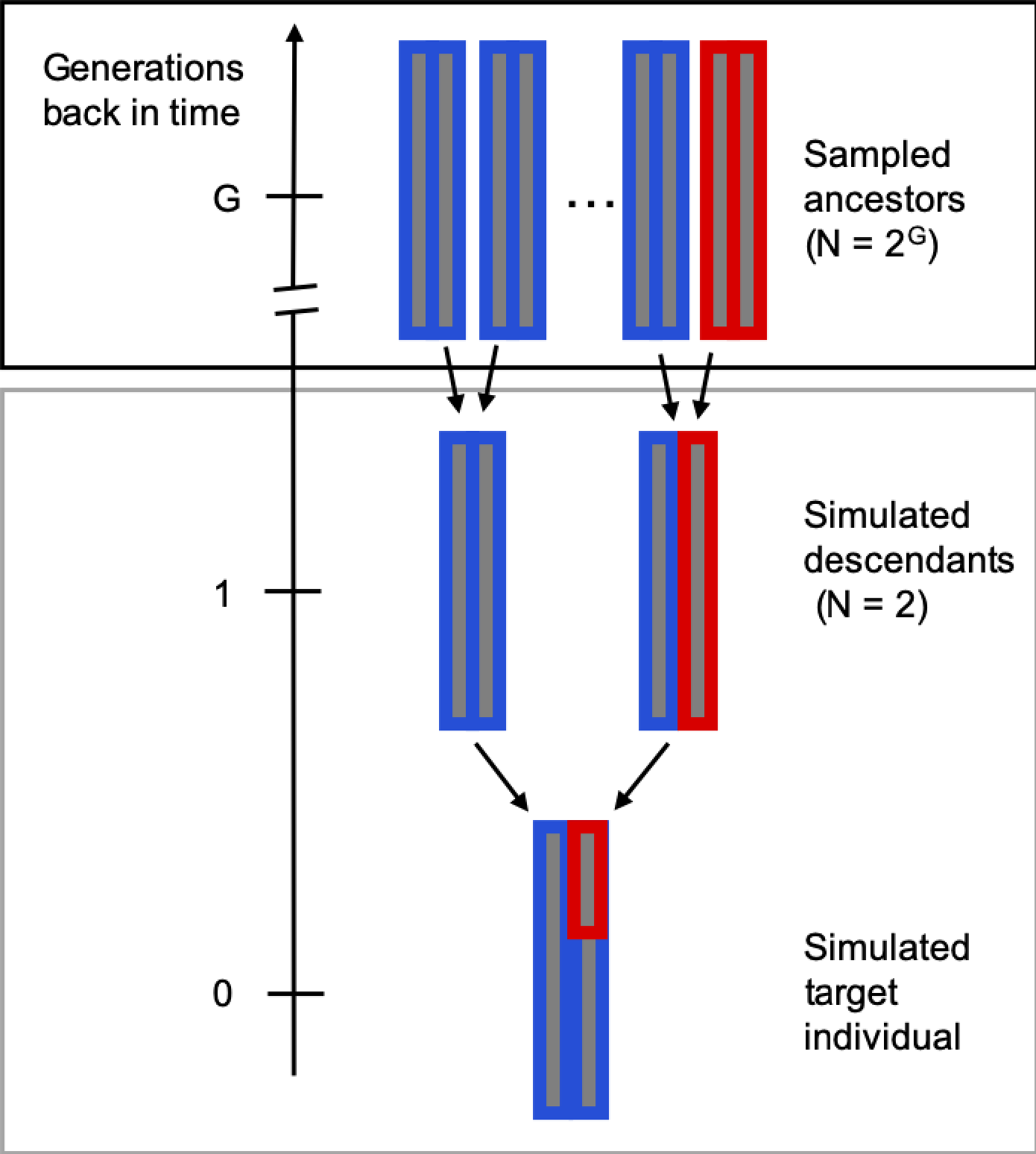

Supplement: S7 Fig — In each simulation, 2G individuals were sampled to represent the ancestors from G generations back in time (black box), where G varied between 1 and 5. All the subsequent descendants in generations G-1, G-2, …, were simulated to determine the genotypes of the target individual at generation 0 (grey box). In this example simulation, 1 ancestor is sampled from A-West (red) and the remaining 2G – 1 ancestors were sampled from A-East (blue). The two adjacent bars correspond to the two haplotypes of an individual and the color corresponds to the ancestor candidate group. (TIF) [file pgen.1009347.s007.tif]

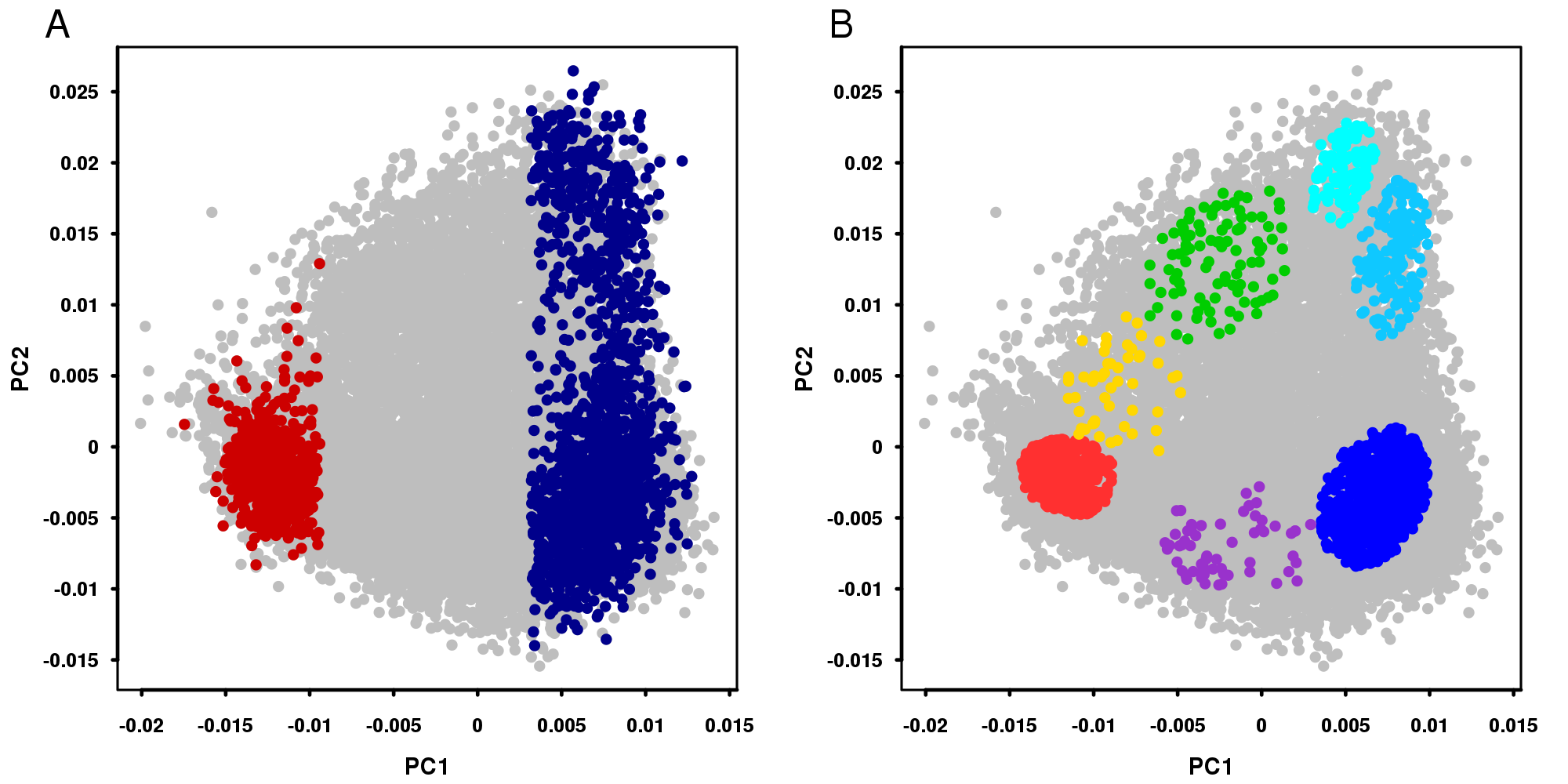

Supplement: S8 Fig — The location of ancestor candidates on a plane defined by principal components (PC) 1 and 2 of the genetic structure for A) simulation settings for refset 2 and B) simulation settings for refsets 6 and 10. (TIF) [file pgen.1009347.s008.tif]

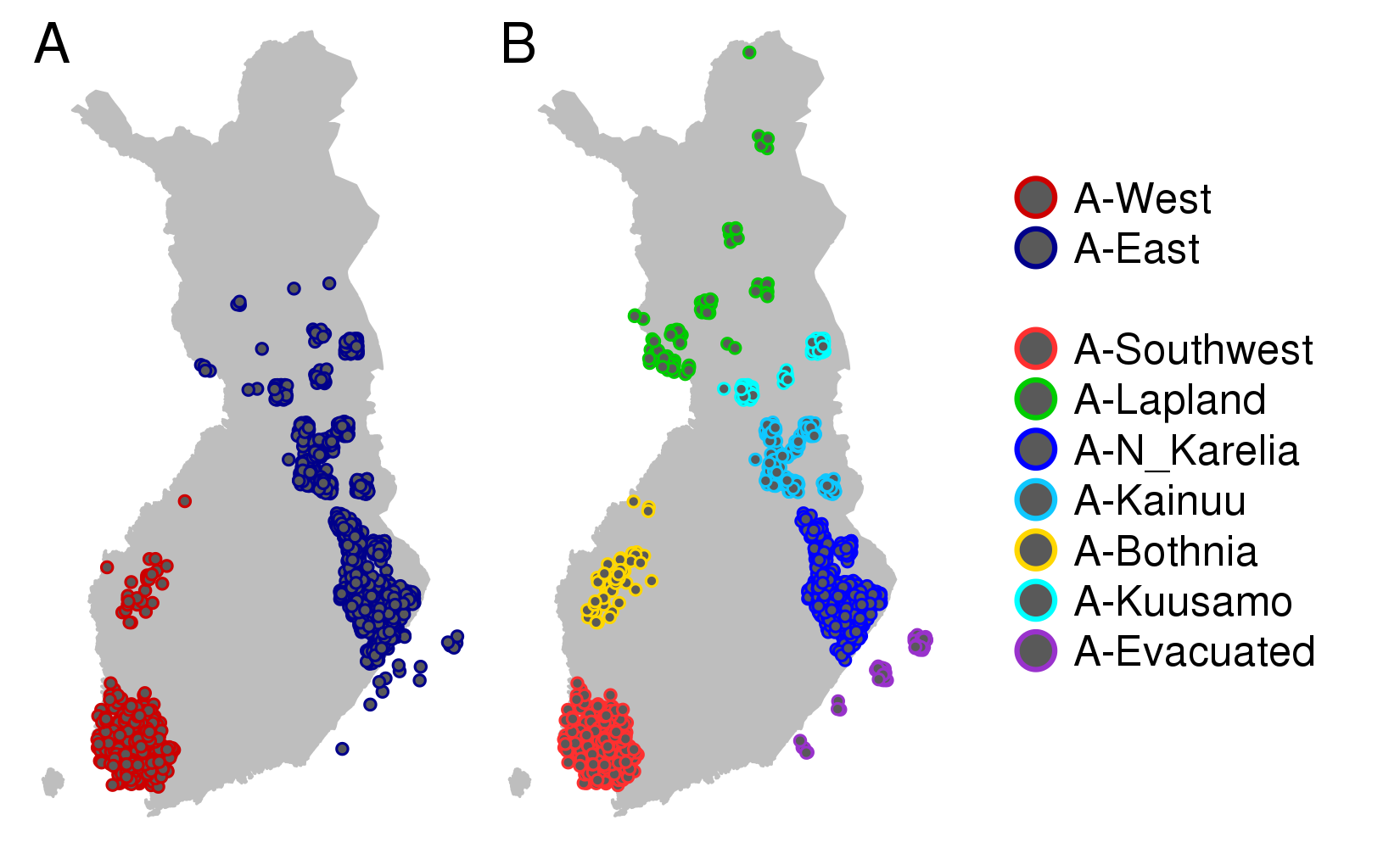

Supplement: S9 Fig — The geographic location of the ancestor candidates in simulation settings A) for refset 2 and B) for refsets 6 and 10. The names of the ancestor candidate groups are shown on right. (TIF) [file pgen.1009347.s009.tif]

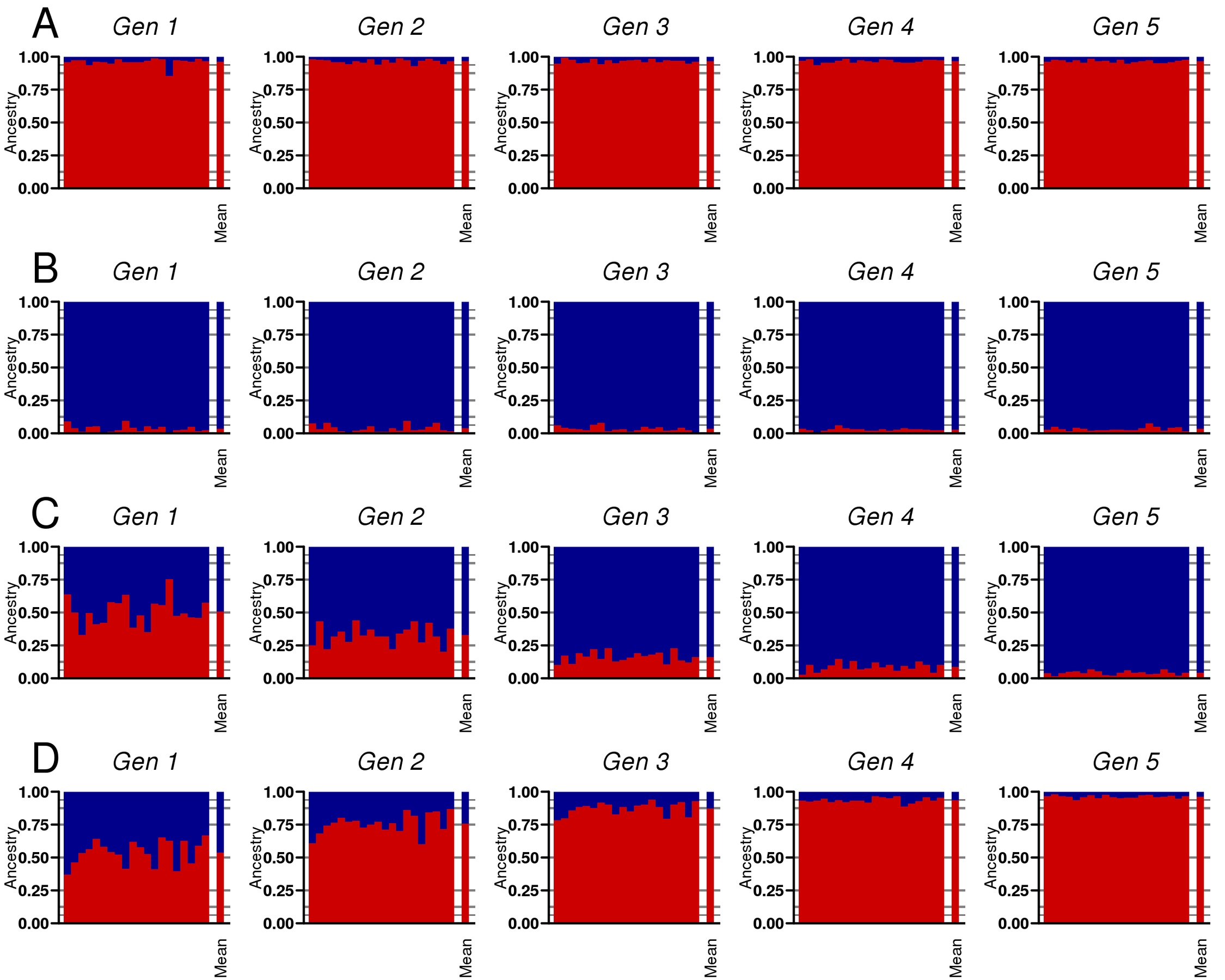

Supplement: S10 Fig — Ancestry profiles for 20 individuals in simulation settings involving ancestry groups R2_East and R2_West: A) for setting All-West, B) for All-East, C) for Almost-East and D) for Almost-West. Blue denotes the estimated proportion in reference group R2_East and red denotes the proportion in reference group R2_West. Gen (1,…,5) refers to the number of generations considered in the simulation. ‘Mean’ shows the average over the 20 simulated individuals. (TIF) [file pgen.1009347.s010.tif]

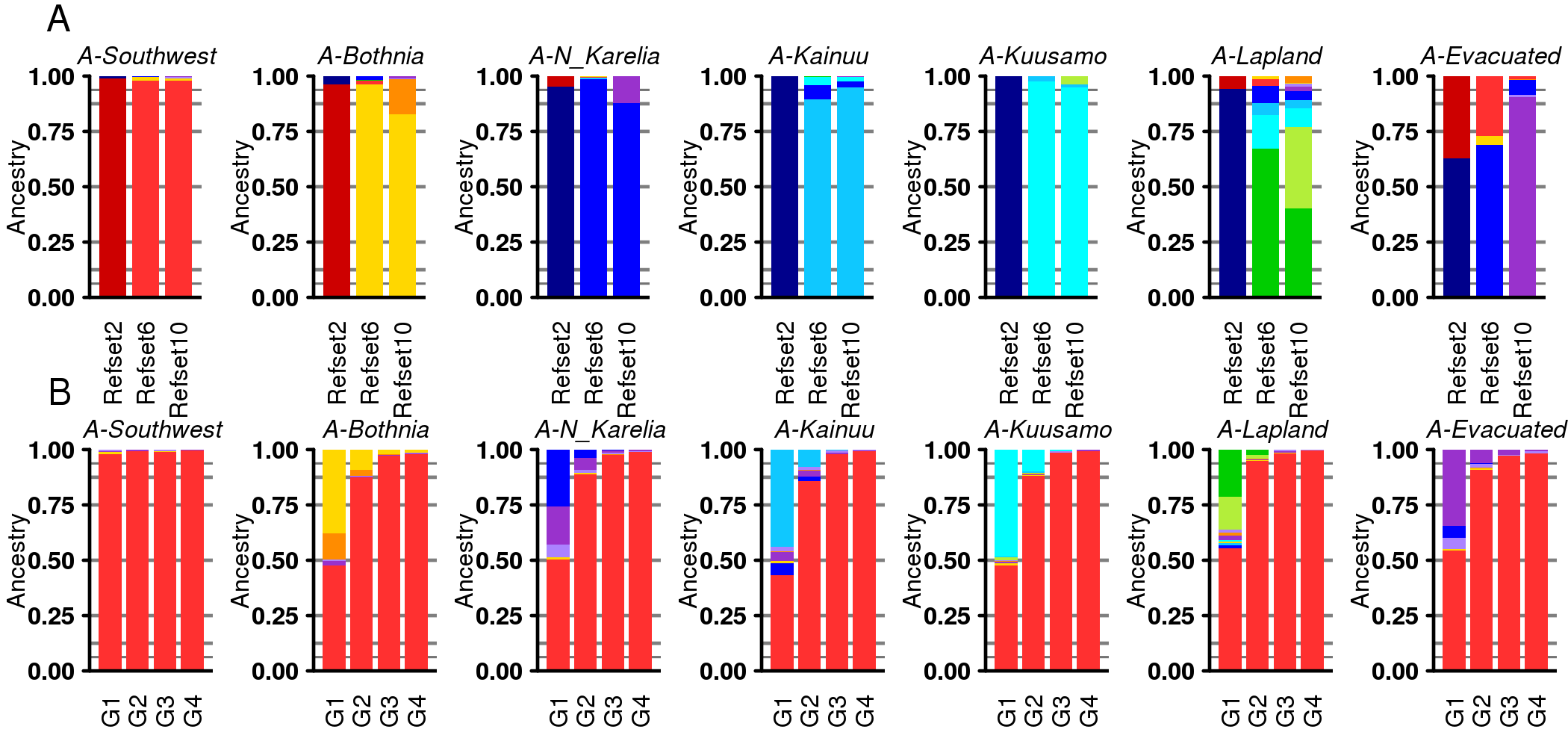

Supplement: S11 Fig — Average ancestry for the simulation results in Fig 4 when the ancestry proportions below 5% were shrunk to zero and the remaining proportions were scaled to one. Panel A) presents individuals whose all ancestors come from one group (single origin) shown in the title estimated using refsets 2, 6 or 10. Panel B) presents individuals whose 2G-1 ancestors, where G = 1…4 is the number of generations, originate from A-Southwest and 1 ancestor originates from the ancestor group in the title, estimated using refset 10. The colors correspond to the reference groups in Fig 2. (TIF) [file pgen.1009347.s011.tif]

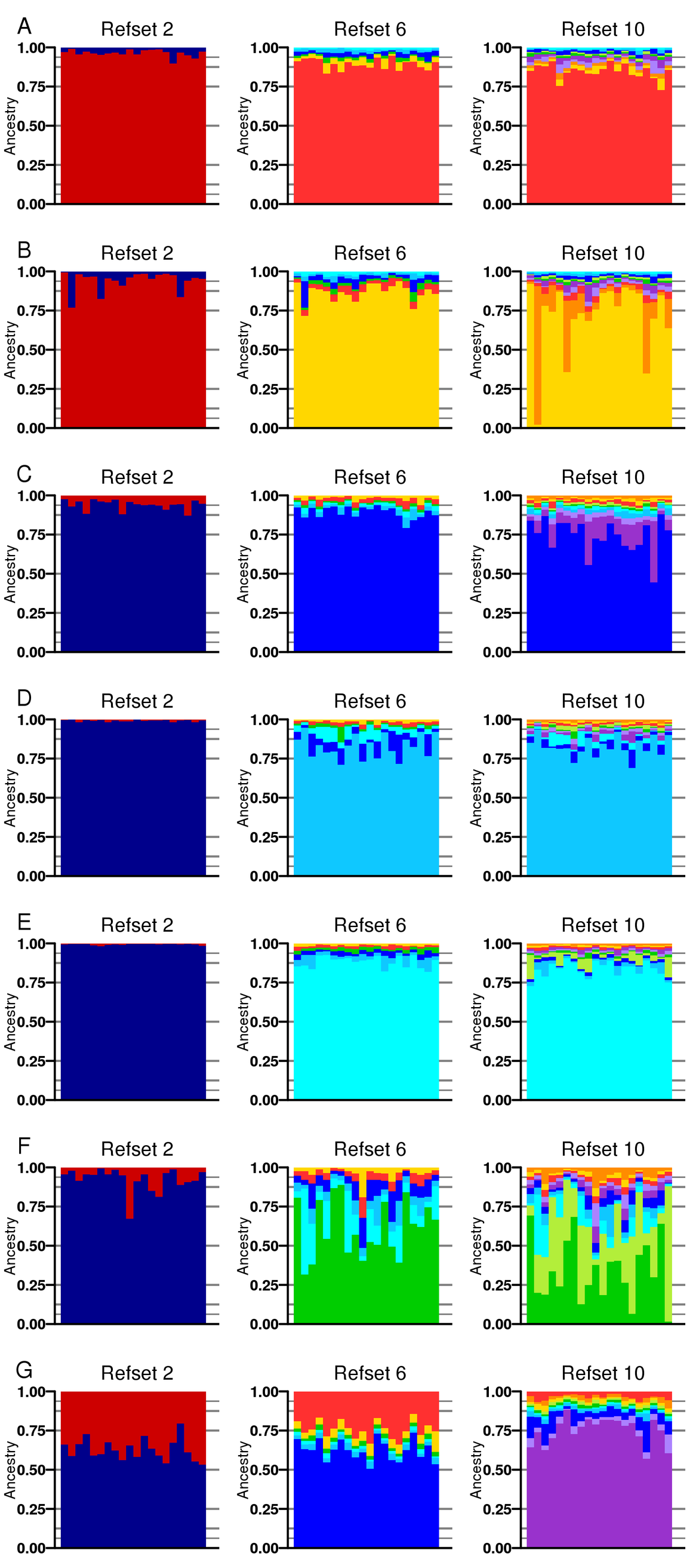

Supplement: S12 Fig — The individual ancestry profiles for 20 individuals whose both parents originate from the ancestor candidate group of A) A-Southwest, B) A-Bothnia, C) A-N_Karelia, D) A-Kainuu, E) A-Kuusamo, F) A-Lapland and G) A-Evacuated (see Fig 4A for the mean values). The colors correspond to the reference groups in Fig 2. (TIF) [file pgen.1009347.s012.tif]

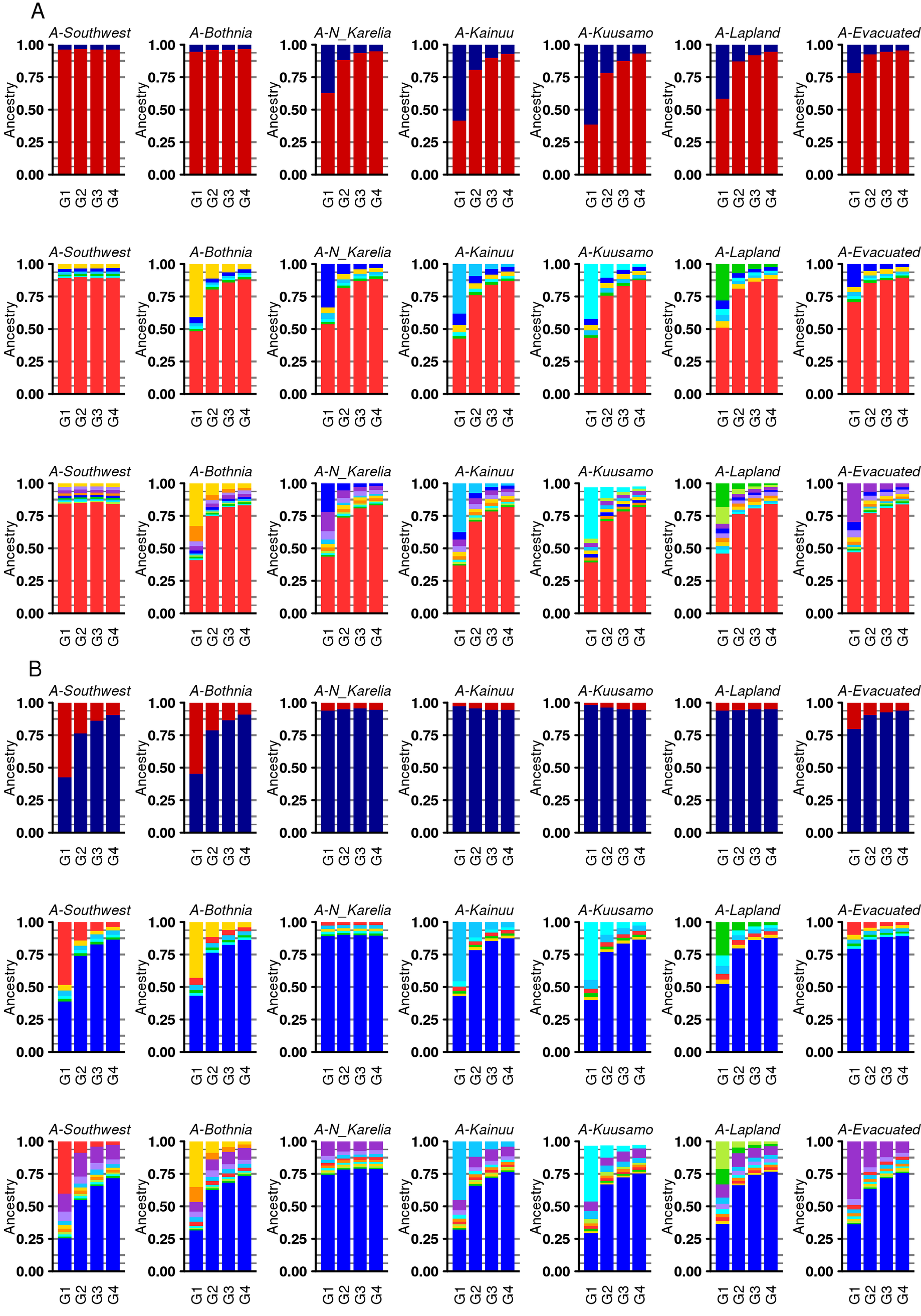

Supplement: S13 Fig — Detailed simulation results for mixed ancestry from ancestor groups A-Southwest and A-N_Karelia. Panel A) presents individuals whose 2G-1, where G is the number of generations, ancestors originate from A-Southwest and 1 ancestor originates from the region in the title. Top row shows the ancestry profiles estimated using refset 2, middle row shows the same for refset 6 and bottom row shows them for refset 10. Panel B) shows the same quantities for a simulation setting where all but one ancestors originate from A-N_Karelia. (TIF) [file pgen.1009347.s013.tif]

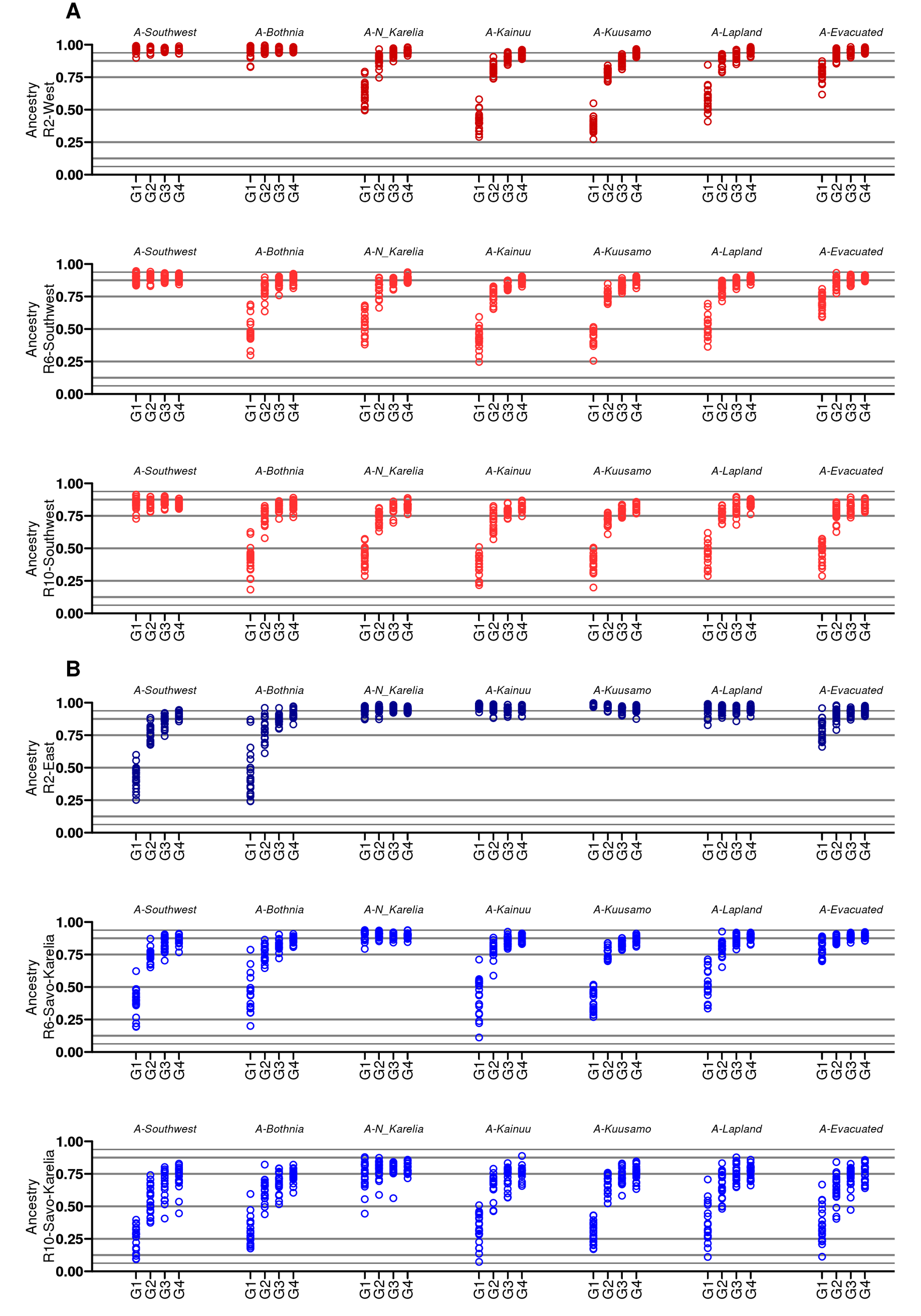

Supplement: S14 Fig — Detailed simulation results for mixed ancestry from ancestor groups A-Southwest and A-N_Karelia (corresponding to S13 Fig). Panel A) presents individuals whose 2G-1, where G is the number of generations, ancestors originate from A-Southwest and 1 ancestor originates from the region in the title. Top row shows estimated ancestry in R2-West, middle row shows the same for R6-Southwest and bottom row shows them for R10-Southwest. Panel B) shows the same quantities for a simulation setting where all but one ancestors originate from A-N_Karelia and the reference groups whose estimates are shown are R2-East (top), R6-Savo-Karelia (middle) and R10-Savo-Karelia (bottom). (TIF) [file pgen.1009347.s014.tif]

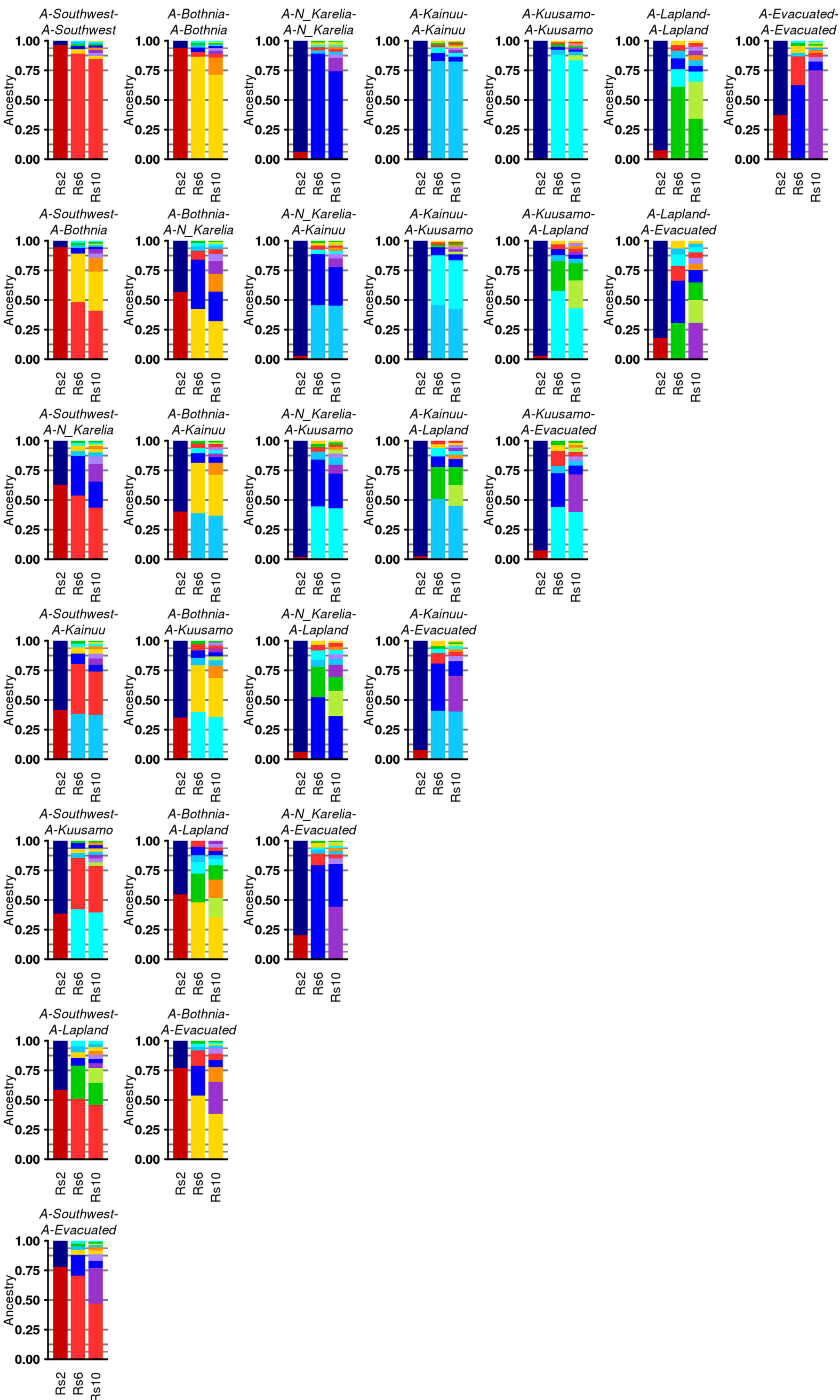

Supplement: S15 Fig — Average ancestry profiles for 20 simulated individuals whose parents come from different geographic regions are shown. Title describes the ancestor candidate groups of the parents used in the simulation. (TIF) [file pgen.1009347.s015.tif]

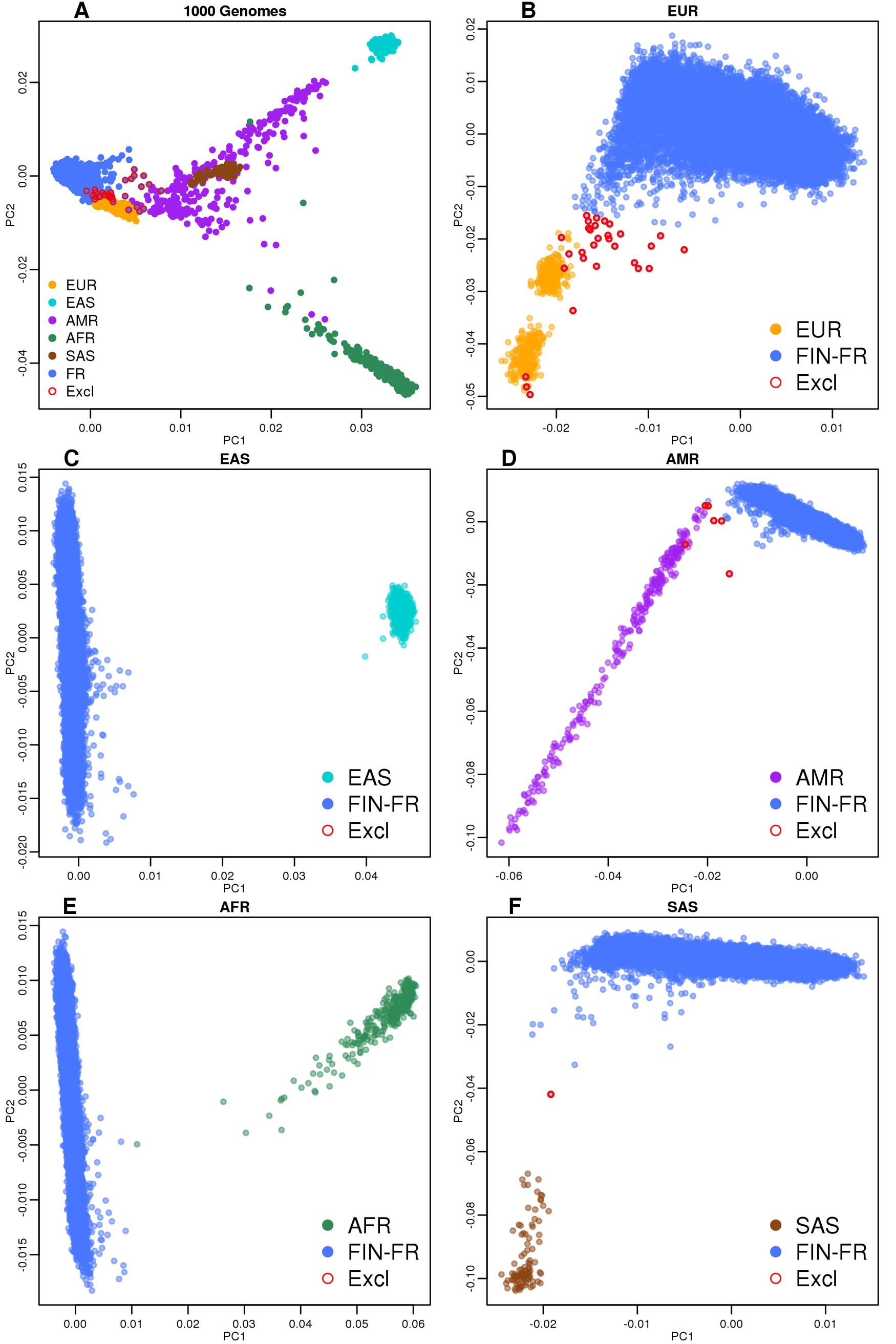

Supplement: S16 Fig — A) PCA of 5 super populations of the 1000 Genomes (Phase 3) samples and our Finnish FINRISK (FIN-FR) samples. PCA of the FINRISK samples together with the B) non-Finnish European (EUR), C) East Asian (EAS), D) American (AMR), E) African (AFR) and F) South Asian (SAS) samples of the 1000 Genomes Phase 3. The FINRISK samples circled with red were identified to show admixture with one or more super populations and were excluded from the regional ancestry analyses. None of our reference individuals was among the excluded. (TIF) [file pgen.1009347.s016.tif]

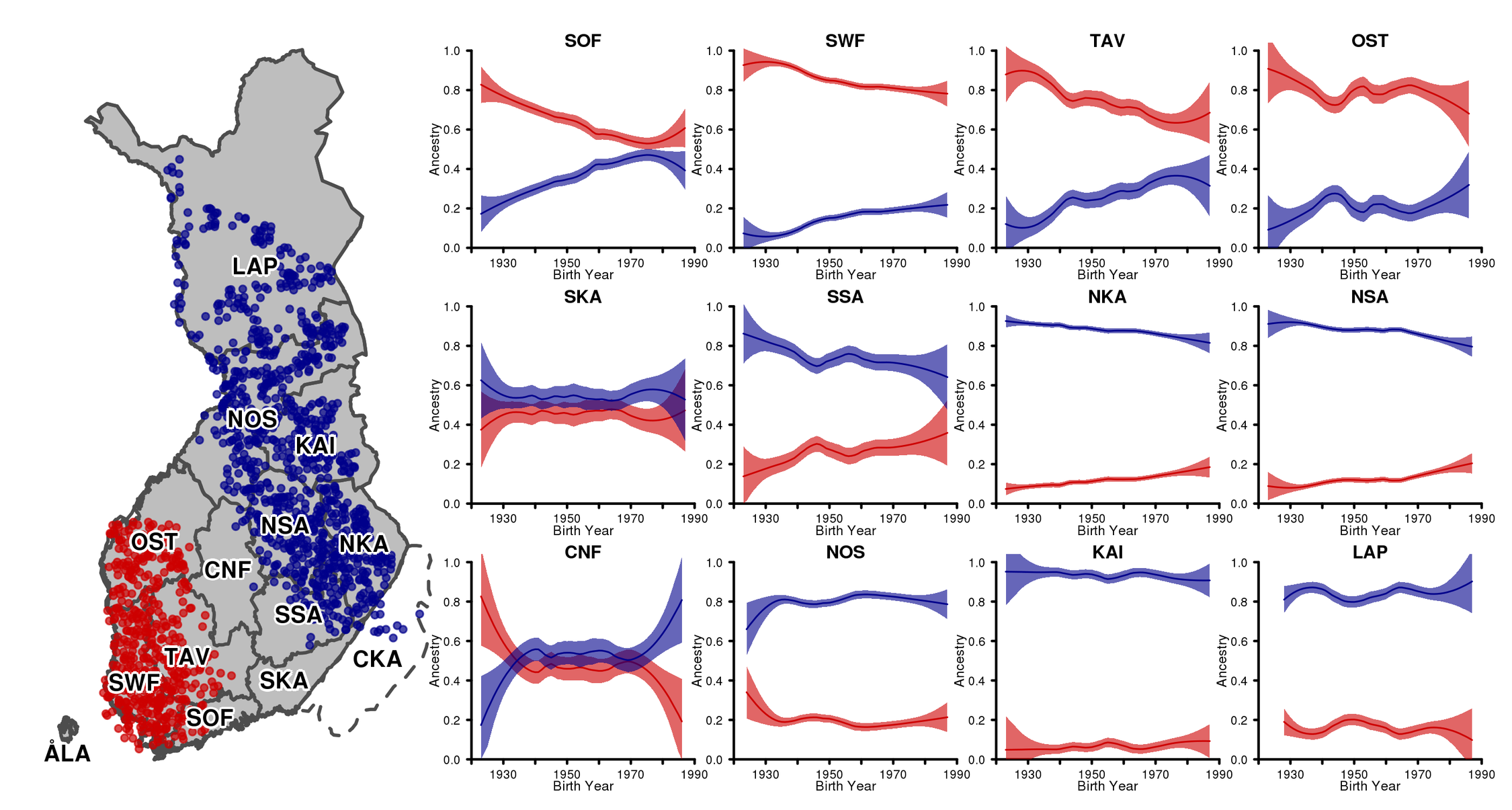

Supplement: S17 Fig — The map on the left shows the regions and the locations of the individuals who form the 2 reference groups. The curves show the estimated ancestry proportion in each reference group as a function of the birth years of individuals born in each region (name of the region in the title). (TIF) [file pgen.1009347.s017.tif]

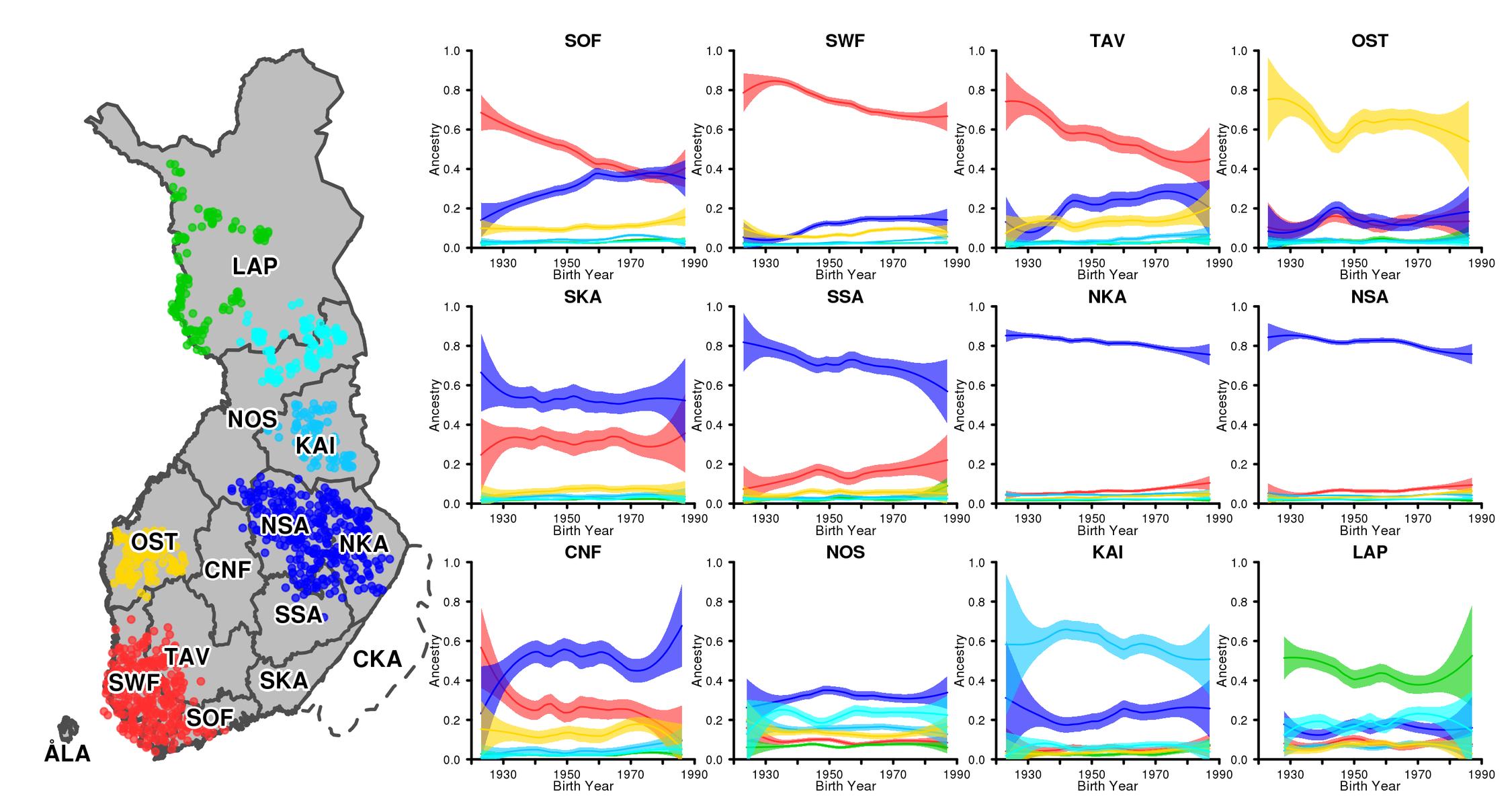

Supplement: S18 Fig — The map on the left shows the regions and the locations of the individuals who form the 6 reference groups. The curves show the estimated ancestry proportion in each reference group as a function of the birth years of individuals born in each region (name of the region in the title). (TIF) [file pgen.1009347.s018.tif]

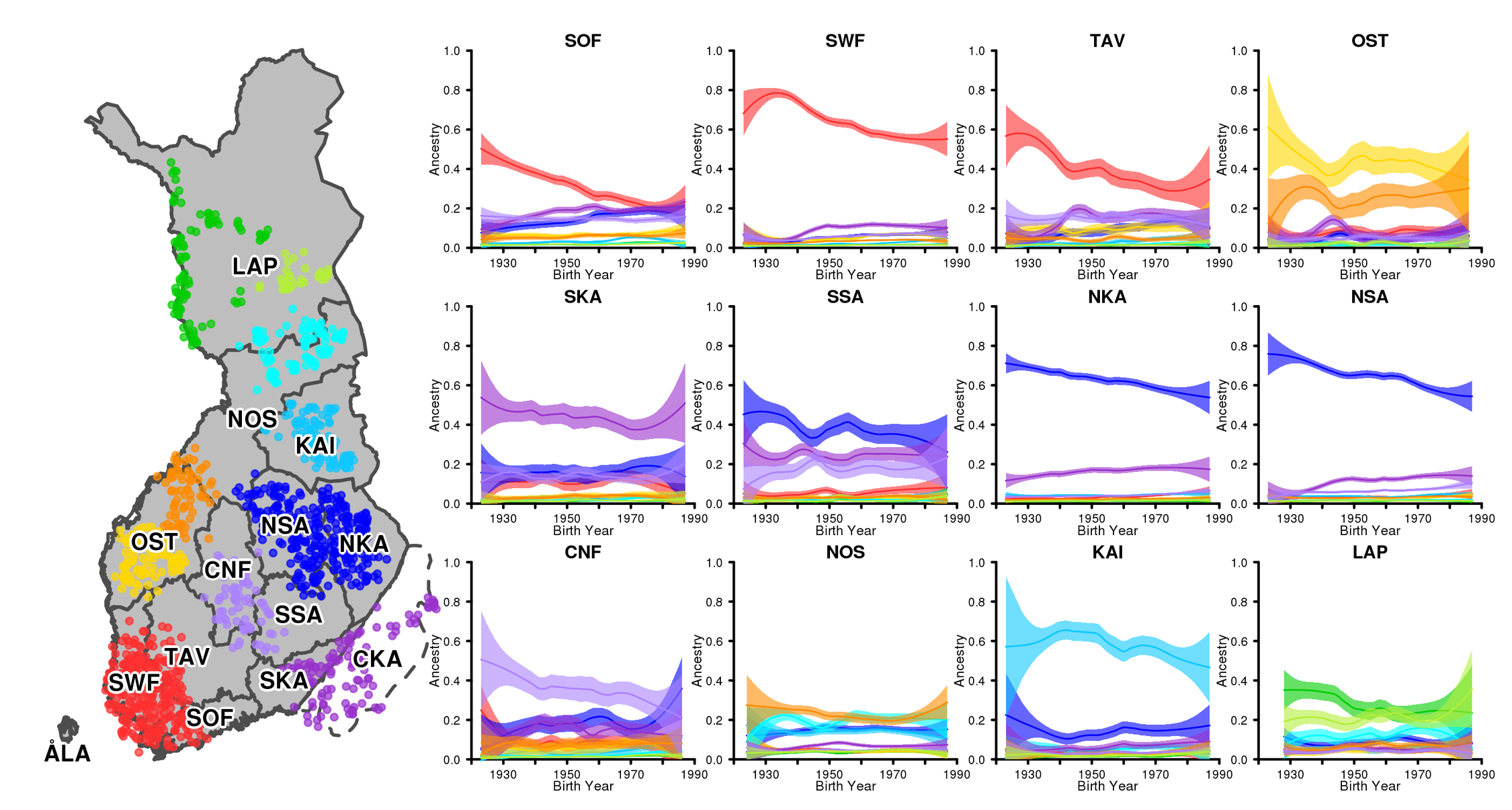

Supplement: S19 Fig — The map on the left shows the regions and the locations of the individuals who form the 10 reference groups. The curves show the estimated ancestry proportion in each reference group as a function of the birth years of individuals born in each region (name of the region in the title). (TIF) [file pgen.1009347.s019.tif]

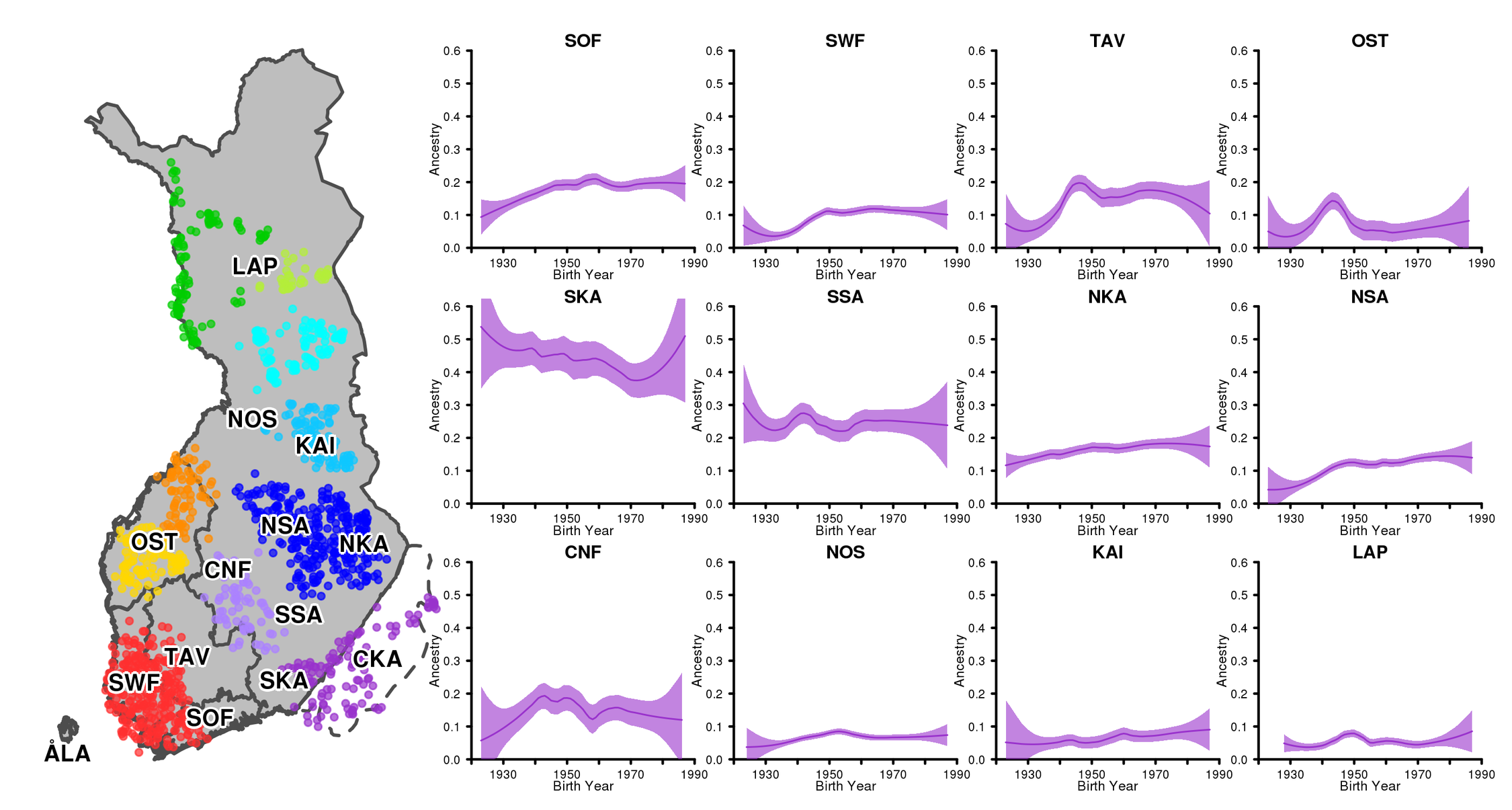

Supplement: S20 Fig — Changes in the genetic ancestry proportion within mainland Finland using refset 10 but showing only the ancestry proportion from R10-Evacuated. The map on the left shows the location of reference individuals and R10-Evacuated is located at southeast corner of the map overlapping the region of Ceded Karelia (CKA). (TIF) [file pgen.1009347.s020.tif]

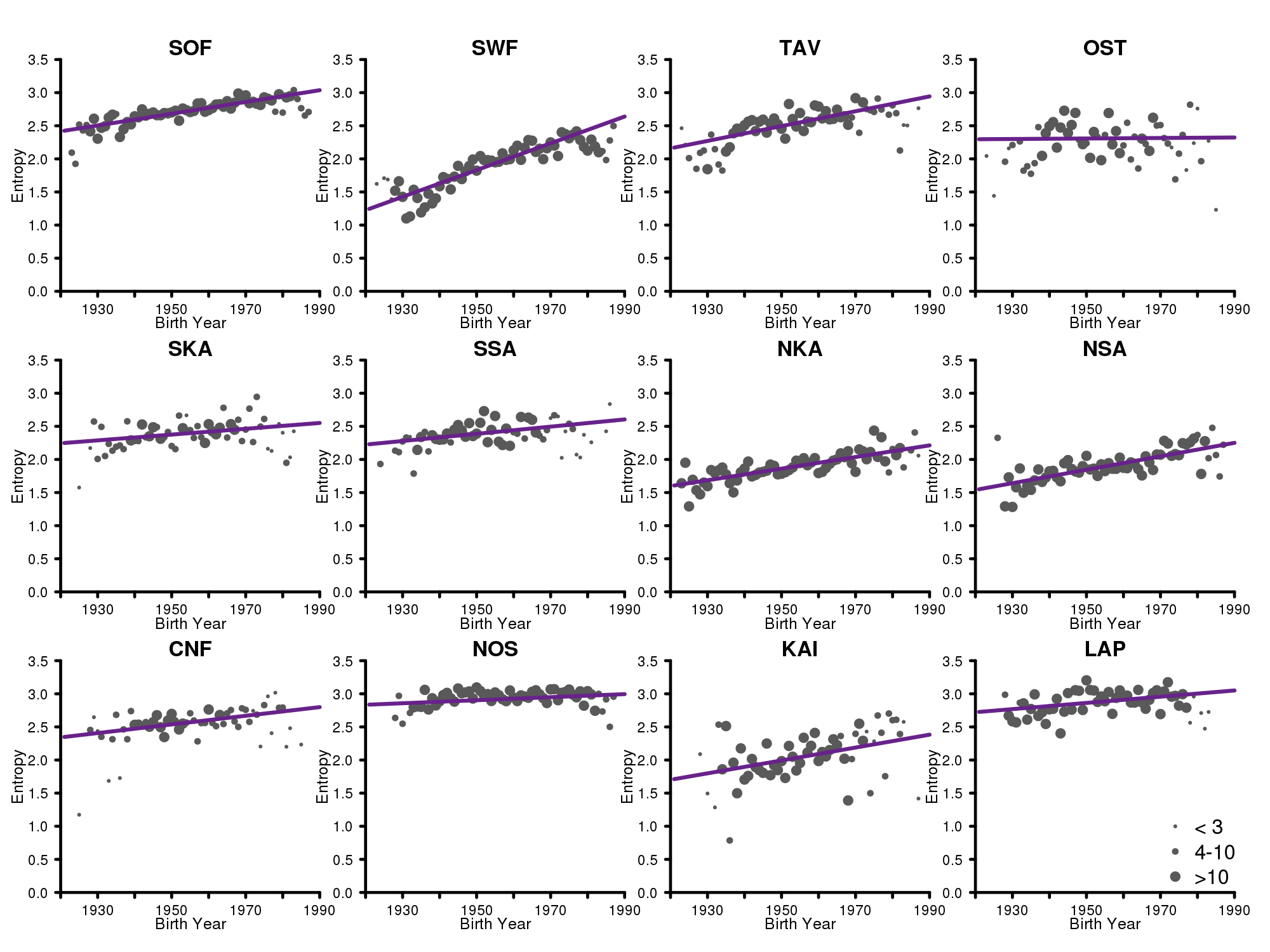

Supplement: S21 Fig — The points represent entropy of the mean profile of individuals born during one year in a particular region estimated with respect to refset 10. The size of the point represents the number of individuals averaged in that yearly profile. The purple line is the linear regression line fitted to the data. The slope estimates the rate of change in heterogeneity of the ancestry profile. (TIF) [file pgen.1009347.s021.tif]

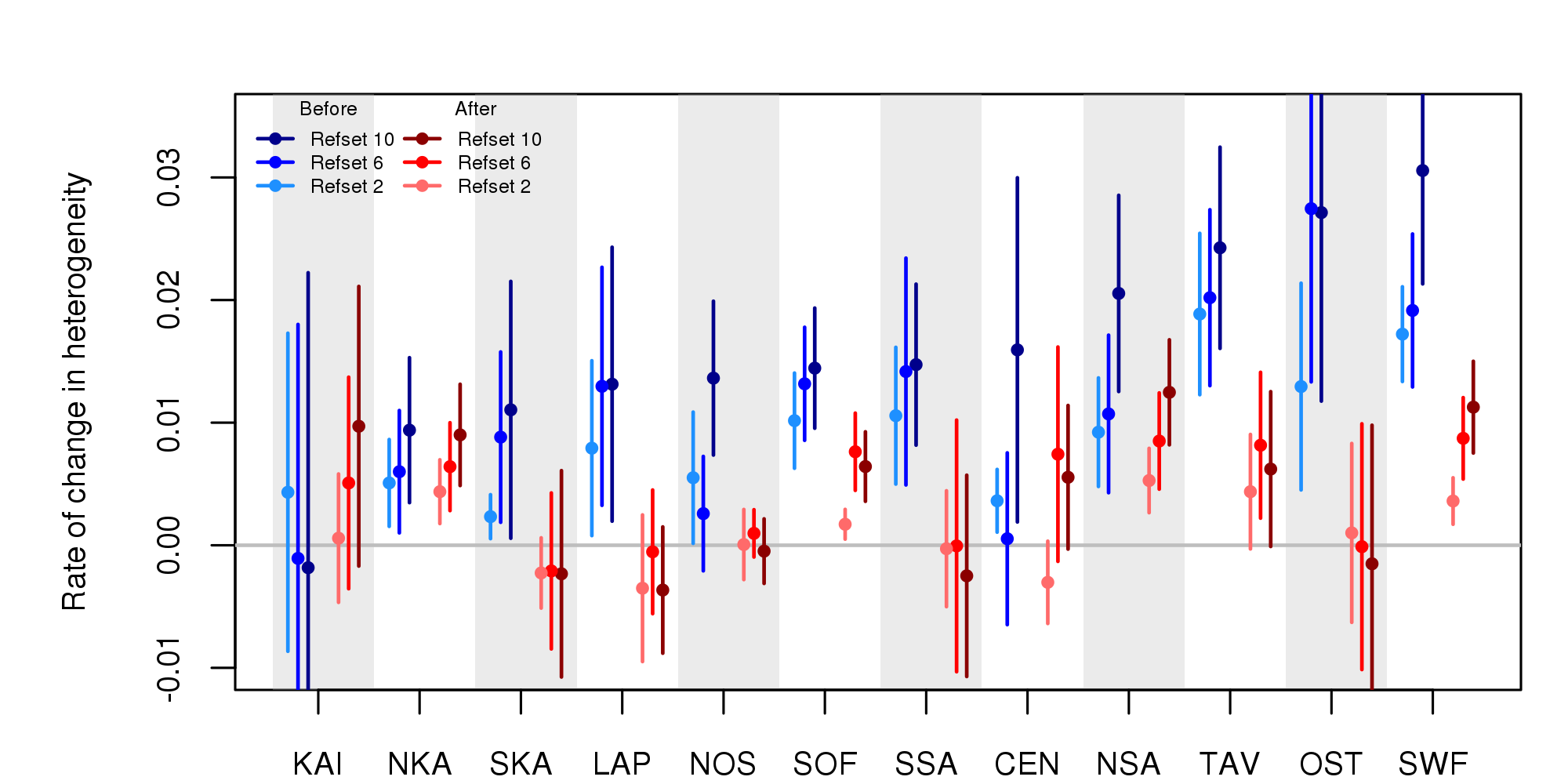

Supplement: S22 Fig — The rate of change is calculated by regressing the mean entropy over the years with refsets 2, 6, and 10 on the year of birth. (TIF) [file pgen.1009347.s022.tif]

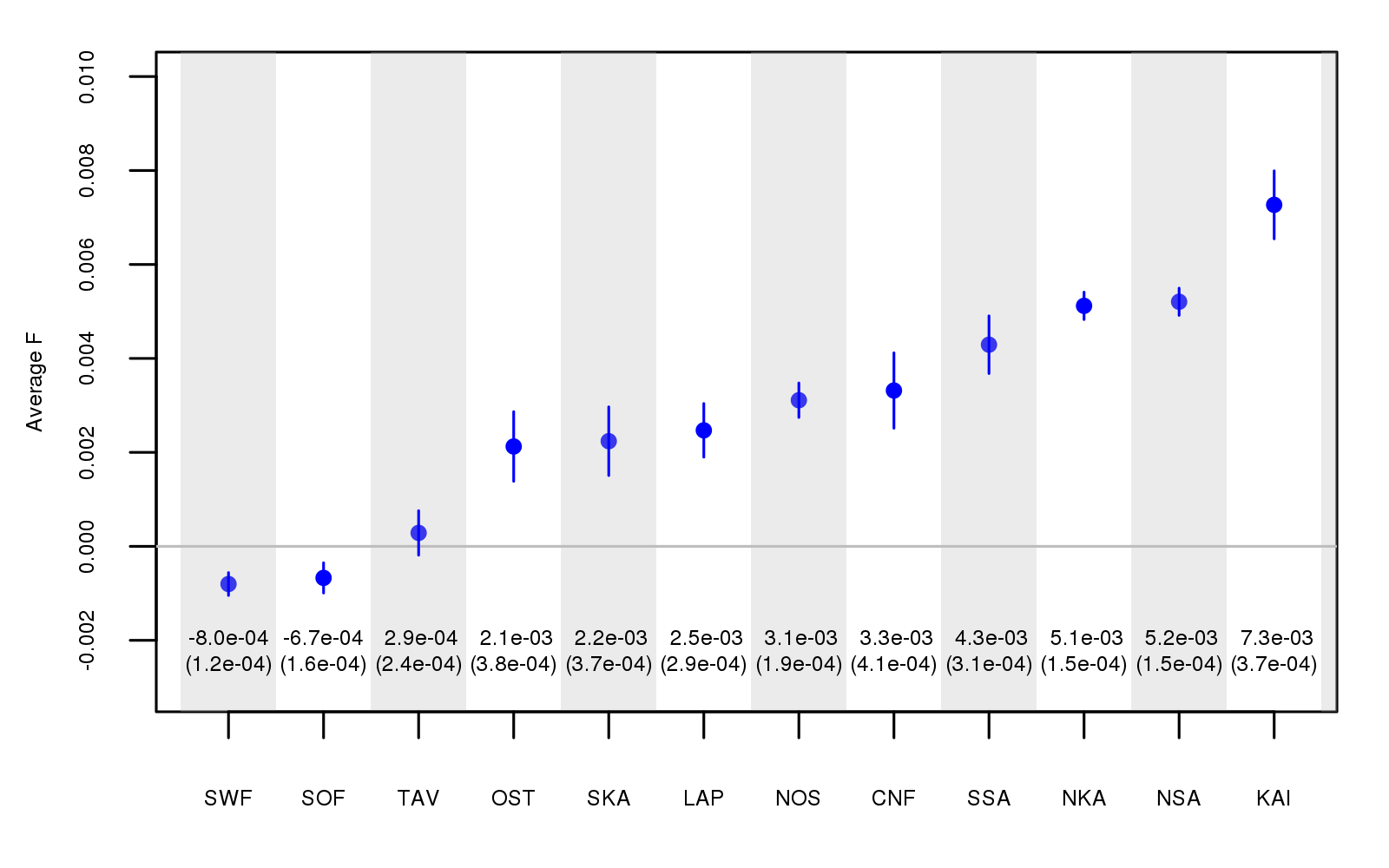

Supplement: S23 Fig — The values at the bottom report the average F per region with its standard error in parentheses. Whiskers show the 95% confidence interval. (TIF) [file pgen.1009347.s023.tif]
