# Supplementary material for: Changes in the fine-scale genetic structure of Finland through the 20th century
Source: PLoS Genet. 2021 Mar 4;17(3):e1009347. doi: 10.1371/journal.pgen.1009347 (PMC7932171; doi:10.1371/journal.pgen.1009347)
Supplement: S1 Table — The number of individuals incorrectly assigned to a single origin, out of 20, based on whether their A) West ancestry component or B) East ancestry component was above the threshold. The threshold was defined as the second largest value in A) All-West or B) All-East simulation setting (corresponding to the 95% quantile in the simulation setting). (PDF) [file pgen.1009347.s024.pdf]

S1 Table. Number of incorrectly assigned individuals in simulations between East and West.

The number of individuals incorrectly assigned to a single origin, out of 20, based on whether their A) West ancestry component or B) East ancestry component was above the threshold. The threshold was defined as the second largest value in A) *All-West* or B) *All-East* simulation setting (corresponding to the 95% quantile in the simulation setting).

	**A West ancestry**		**B East ancestry**	
**Generation**	***Almost-East***	***Almost-West***	***Almost-East***	***Almost-East***
1	0	0	0	0
2	0	0	0	0
3	0	0	0	0
4	0	5	1	0
5	0	18	15	0
