# Supplementary material for: Changes in the fine-scale genetic structure of Finland through the 20th century
Source: PLoS Genet. 2021 Mar 4;17(3):e1009347. doi: 10.1371/journal.pgen.1009347 (PMC7932171; doi:10.1371/journal.pgen.1009347)
Supplement: S2 Table — (PDF) [file pgen.1009347.s025.pdf]

S2 Table. Pairwise-F_{ST} values ($\times 10^5$) between ancestor candidate groups and reference groups of refset 10 (lower triangular) and their standard errors (upper triangular).

	Ancestor groups							Reference groups (Level 10)									
	A-Southwest	A-West_Lapland	A-N_Karelia	A-Kainuu	A-Bothnia	A-Kuusamo	A-Evacuated	R10-Southwest	R10-East Lapland	R10-West_Lapland	R10-Savo-Karelia	R10-Kainuu	R10-Bothnia	R10-Kuusamo	R10-Evacuated	R10-Kokkola	R10-Central_Finland
**A-Southwest**	-	4	4	6	6	7	6	1	11	6	5	8	5	7	4	5	6
**A-West_Lapland**	271	-	5	7	9	7	8	5	11	6	6	8	7	7	6	8	8
**A-N_Karelia**	366	262	-	3	8	5	5	4	10	6	1	5	7	5	3	7	5
**A-Kainuu**	530	383	209	-	10	6	8	7	12	8	4	4	9	6	6	8	7
**A-Bothnia**	227	333	406	565	-	11	10	7	14	10	9	11	7	11	8	8	10
**A-Kuusamo**	531	367	305	326	570	-	9	8	12	9	6	8	9	5	7	10	9
**A-Evacuated**	282	308	242	428	396	471	-	7	14	9	6	9	9	10	6	9	9
**R10-Southwest**	6	266	360	522	222	524	278	-	12	6	5	8	5	8	5	5	6
**R10-East_Lapland**	554	229	490	596	615	523	572	557	-	13	11	13	14	12	11	14	14
**R10-West_Lapland**	379	162	466	605	457	589	472	369	497	-	7	10	8	9	7	9	9
**R10-Savo-Karelia**	409	286	26	201	440	302	296	404	514	497	-	5	8	6	4	7	6
**R10-Kainuu**	558	408	235	8	579	358	453	546	618	632	219	-	10	8	7	10	8
**R10-Bothnia**	255	385	462	619	33	624	425	251	661	505	498	637	-	9	7	8	8
**R10-Kuusamo**	545	374	305	336	574	57	481	538	538	602	302	363	639	-	7	10	9
**R10-Evacuated**	248	254	150	338	333	385	118	238	503	415	185	362	371	392	-	7	6
**R10-Kokkola**	269	328	384	526	214	534	390	267	582	472	405	542	338	544	343	-	9
**R10-Central_Finland**	275	299	212	357	356	414	300	268	545	454	226	380	397	423	160	367	-
