# Supplementary material for: Changes in the fine-scale genetic structure of Finland through the 20th century
Source: PLoS Genet. 2021 Mar 4;17(3):e1009347. doi: 10.1371/journal.pgen.1009347 (PMC7932171; doi:10.1371/journal.pgen.1009347)
Supplement: S3 Table — Column ‘Expected ancestry’ shows which reference groups were considered the closest to the corresponding ancestor group, and the ‘Total expected ancestry’ sums over the expected ancestries. Unexpected ancestry was defined as everything else except the expected and the average contribution of those groups is shown in column ‘Average unexpected ancestry’. Column ‘No shrink’ shows the values of the raw ancestry estimates and column ‘Shrink <5%’ shows the results after shrinking the individual ancestry estimates below 5% to zero and rescaling the remaining non-zero ancestry proportions back to 100%. (PDF) [file pgen.1009347.s026.pdf]

S3 Table. Ancestry proportions for single-origin simulations categorized into expected and unexpected ancestry.

Column 'Expected ancestry' shows which reference groups were considered the closest to the corresponding ancestor group, and the 'Total expected ancestry' sums over the expected ancestries. Unexpected ancestry was defined as everything else except the expected and the average contribution of those groups is shown in column 'Average unexpected ancestry'. Column 'No shrink' shows the values of the raw ancestry estimates and column 'Shrink <5%' shows the results after shrinking the individual ancestry estimates below 5% to zero and rescaling the remaining non-zero ancestry proportions back to 100%.

		**Total expected ancestry**		**Average unexpected ancestry**	
**Single-origin ancestor group**	**Expected ancestry**	**No shrink**	**Shrink <5%**	**No shrink**	**Shrink <5%**
Refset 6					
A-Southwest	R6-Southwest	0.892	0.980	0.022	0.004
A-Bothnia	R6-Bothnia	0.865	0.963	0.027	0.007
A-N_Karelia	R6-Savo-Karelia	0.891	0.986	0.022	0.003
A-Kainuu	R6-Kainuu	0.826	0.896	0.035	0.021
A-Kuusamo	R6-Kuusamo	0.882	0.974	0.024	0.005
A-Lapland	R6-West_Lapland	0.611	0.672	0.078	0.066
A-Evacuated	R6-Savo-Karelia	0.624	0.690	0.075	0.062
Refset 10					
A-Southwest	R10-Southwest	0.846	0.979	0.017	0.002
A-Bothnia	R10-Bothnia, R10-Kokkola	0.859	0.987	0.018	0.002
A-N_Karelia	R10-Savo-Karelia	0.742	0.880	0.029	0.013
A-Kainuu	R10-Kainuu	0.826	0.950	0.019	0.006
A-Kuusamo	R10-Kuusamo	0.833	0.950	0.019	0.006
A-Lapland	R10-West_Lapland, R10-East_Lapland	0.655	0.769	0.043	0.029
A-Evacuated	R10-Evacuated	0.749	0.904	0.028	0.011
