# Supplementary material for: Changes in the fine-scale genetic structure of Finland through the 20th century
Source: PLoS Genet. 2021 Mar 4;17(3):e1009347. doi: 10.1371/journal.pgen.1009347 (PMC7932171; doi:10.1371/journal.pgen.1009347)
Supplement: S4 Table — Mann-Whitney p-value corresponds to a test between the focal group and the union of the rest of the groups at that refset. (PDF) [file pgen.1009347.s027.pdf]

S4 Table. Range of the birth years (Min and Max) and the mean birth years of the reference groups.

Mann-Whitney p-value corresponds to a test between the focal group and the union of the rest of the groups at that refset.

	Min	Max	Mean	Mann-Whitney p-value
Refset 2				
R2-West	1923	1987	1953	0.09
R2-East	1923	1987	1954	0.09
Refset 6				
R6-Southwest	1924	1983	1952	0.05
R6-West_Lapland	1928	1987	1952	0.28
R6-Savo_Karelia	1923	1986	1953	0.68
R6-Kainuu	1933	1985	1953	0.68
K6-Bothnia	1923	1985	1956	0.04
R6-Kuusamo	1928	1984	1955	0.07
Refset 10				
R10-Southwest	1924	1983	1951	0.18
R10-West_Lapland	1928	1977	1952	0.62
R10-Savo_Karelia	1924	1986	1953	0.40
R10-Kainuu	1933	1985	1953	0.59
K10-Bothnia	1925	1985	1955	0.03
R10-Kuusamo	1928	1984	1955	0.02
R10-Evacuated	1925	1981	1946	4.3E-07
R10-Kokkola	1932	1981	1953	0.72
R10-Central_Finland	1925	1987	1954	0.49
R10-East_Lapland	1930	1973	1955	0.18
Ancestor candidates				
A-Southwest	1926	1987	1954	0.01
A-Lapland	1928	1982	1954	0.12
A-N_Karelia	1923	1987	1951	0.06
A-Kainuu	1930	1987	1954	0.18
A-Bothnia	1928	1978	1951	0.49
A-Kuusamo	1933	1985	1954	0.44
A-Evacuated	1924	1967	1942	1.7E-09
