# Supplementary material for: Changes in the fine-scale genetic structure of Finland through the 20th century
Source: PLoS Genet. 2021 Mar 4;17(3):e1009347. doi: 10.1371/journal.pgen.1009347 (PMC7932171; doi:10.1371/journal.pgen.1009347)
Supplement: S5 Table — (PDF) [file pgen.1009347.s028.pdf]

S5 Table. **Number of study individuals excluded or included after each filtering step.**

Processing steps	Excluded	Included
Starting number of samples	---	23431
Variant-missigness > 0.005, lHeteroz.l > 0.04	493	22938
Samples on bad quality genotyping plates	138	22800
Born abroad	446	22354
3rd degree relatives	3635	18719
Chr 21 missigness > 0.1	4	18715
Missing birth municipality	216	18499
Born in municipality of Karjala	5	18494
1000G PCA outliers	31	18463
