# Supplementary material for: Changes in the fine-scale genetic structure of Finland through the 20th century
Source: PLoS Genet. 2021 Mar 4;17(3):e1009347. doi: 10.1371/journal.pgen.1009347 (PMC7932171; doi:10.1371/journal.pgen.1009347)
Supplement: S6 Table — The numbers in parentheses refer to the number of populations excluded or included. (PDF) [file pgen.1009347.s029.pdf]

S6 Table. Number of reference candidates excluded or included after the steps of reference group identification process.

The numbers in parentheses refer to the number of populations excluded or included.

	Refset 2						Refset 6						Refset 10					
**Step**	**Threshold**	**Exlcuded**		**Remained**		**Threshold**	**Exlcuded**		**Remained**		**Threshold**	**Exlcuded**		**Remained**				
1. Starting level				2741	(2)				2741	(15)				2741	(15)			
2. Initial ancestry																		
3. Identity proportions																		
4. Population exclusions	< 0.50	0	(0)	2741	(2)	< 0.50	1081	(5)	1660	(10)	< 0.50	1081	(5)	1660	(10)			
						< 0.70	560	(4)	1100	(6)								
5. Candidate exclusions	< 0.95	1266	(0)	1475	(2)	< 0.70	63	(0)	1037	(6)	< 0.70	408	(0)	1252	(10)			
6. Geographic outliers		3	(0)	1472	(2)		11	(0)	1027	(6)		16	(0)	1236	(10)			
