# Supplementary material for: Changes in the fine-scale genetic structure of Finland through the 20th century
Source: PLoS Genet. 2021 Mar 4;17(3):e1009347. doi: 10.1371/journal.pgen.1009347 (PMC7932171; doi:10.1371/journal.pgen.1009347)
Supplement: Attachment — Submitted filename: Responses_PlosGen_review_round1_v5.pdf [file pgen.1009347.s030.pdf]

Responses to the editor and reviewers' comments

Editor

(Line numbers refer to the marked-up copy of the manuscript.)

Dear Dr Pirinen,

Both reviewers appreciated the attention to an important problem, and the relevance of the analyses.

We thank the reviewers for their useful comments and the editor for the opportunity to revise our work for PLoS Genetics.

Both reviewers also expressed some confusion about the clustering and filtering steps, and I think that this would be important to address. Perhaps a graphical representation, as suggested by Reviewer 2, would be helpful.

To clarify the analysis steps and simulation scenarios, we have made the following changes to the manuscript:

- Added a workflow of our study (S1 Fig) to the supporting information.
- Line 166: Added a sentence referring to S1 Fig: "S1 Fig *describes the workflow of the study.*"
- Added a graphical presentation of simulation scenarios (S7 Fig) to the supporting information.
- Lines 334-340: Added the following paragraph describing our simulation scenarios:

  "We tested the identifiability of ancestry from different reference groups using simulations where 2^G individuals were sampled to represent the ancestors from G generations back in time (G varied between 1 and 5). We simulated the meioses within these ancestors, and within their subsequent descendants in generations G-1, G-2, …, 1, to determine the genotypes of the target individual at generation 0. The ancestry of the target individual was then estimated and compared to the expected ancestry groups of the sampled ancestors based on their geographic and genetic origin (see S7 Fig for a schematic representation)."

- Lines 194-219: Named the steps in the reference group identification process to ease referring to those steps, e.g., when describing the number of reference candidates excluded. We also made a few grammatical corrections to the steps.

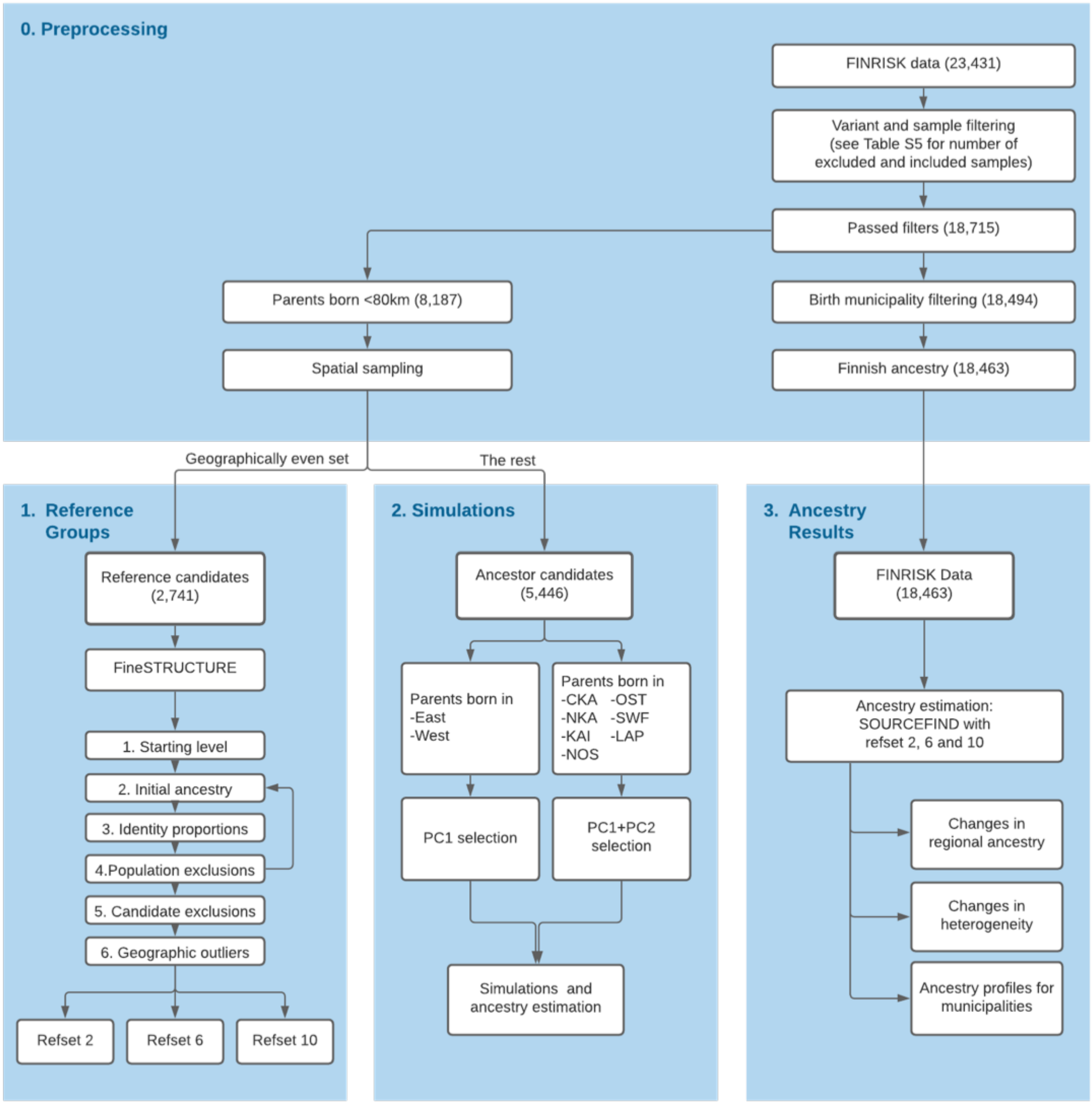

S1 Fig. Workflow of the study.

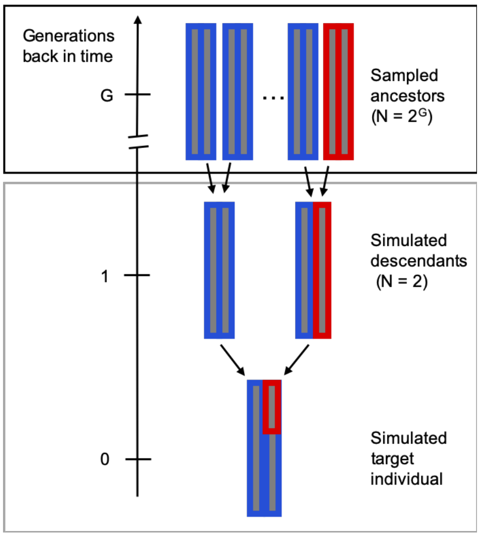

S7 Fig. A schematic representation of our simulation strategy. (See full caption in the responses to minor comments of Reviewer2).

The steps of identification of the reference groups:

1. **Starting level**
   Choose K, the starting number of FineSTRUCTURE populations from the FS-tree.
2. **Initial ancestry**
   Estimate the genetic ancestry of the reference candidates with respect to the K populations using SOURCEFIND.
3. **Identity proportions**
   For each of the K populations, calculate the population's *identity proportion* as the average proportion of ancestry in that particular population across the individuals assigned to that population by FineSTRUCTURE.
4. **Population exclusions**
   Exclude the populations with low identity proportions (< 50%) from the reference candidates, decrease K accordingly, and repeat from step 2. If no population is excluded, proceed to step 5.
5. **Candidate exclusions**
   Exclude the reference candidates who show low levels of ancestry from the population they were assigned to by FineSTRUCTURE. (Thresholds used either < 70% or < 95%.)
6. **Geographic outliers**
   Exclude possible geographic outliers manually (S4 Fig).

I would find it also important to report the number of participants excluded through each filtering step. E.g., on line 206, the manuscript lists the number of outliers, but not the proportion of individuals excluded for low identity proportions. This is important to ascertain how representative the analysis is the the actual population of Finland (see also point 2 of reviewer 2).

We have added tables describing the number of study individuals excluded or included after each filtering steps (S5 Table) and after the steps of identification of reference groups (S6 Table) to the supporting information.

The following changes were made to the manuscript:

- Line 884: Added a reference to S5 Table: "*S5 Table shows the number of excluded or included samples after each filtering step."*
- Line 262: Added a reference to S6 Table: "*S6 Table shows the number of excluded or included reference candidates."*
- Line 870: Corrected the number of excluded individuals in step 3rd degree relatives from 3,677 into 3,635.
- Line 1004: Updated the number of samples in Finnish PCA to include 31 samples excluded as part of 1000 Genomes PCA outliers:

  "*These data included 225 individuals who were not part of the haplotype-based analyses as they were only later excluded due to ambiguous or missing location data.*" →

  "*These data included **256** individuals who were not part of the haplotype-based analyses as they were only later excluded due to ambiguous or missing location data **or as 1000 Genomes PCA outliers (S16 Fig).**"*

S5 Table. Number of study individuals excluded or included after each filtering step.

Processing steps	Excluded	Included
Starting number of samples	---	23431
Variant-missingness > 0.005, IHeteroz.I > 0.04	493	22938
Samples on bad quality genotyping plates	138	22800
Born abroad	446	22354
3rd degree relatives	3635	18719
Chr 21 missigness > 0.1	4	18715
Missing birth municipality	216	18499
Born in municipality of Karjala	5	18494
1000G PCA outliers	31	18463

S6 Table. Number of reference candidates excluded or included after the steps of reference group identification process.

Numbers in parentheses refer to the number of populations excluded or included.

	Refset 2			Refset 6			Refset 10		
Step	Threshold	Excluded	Included	Threshold	Excluded	Included	Threshold	Excluded	Included
1. Starting level			2741 (2)			2741 (15)			2741 (15)
2. Initial ancestry									
3. Identity proportions									
4. Population exclusions	< 0.50	0 (0)	2741 (2)	< 0.50	1081 (5)	1660 (10)	< 0.50	1081 (5)	1660 (10)
				< 0.70	560 (4)	1100 (6)			
5. Candidate exclusions	< 0.95	1266 (0)	1475 (2)	< 0.70	63 (0)	1037 (6)	< 0.70	408 (0)	1252 (10)
6. Geographic outliers		3 (0)	1472 (2)		11 (0)	1026 (6)		16 (0)	1236 (10)

On line 241, it was unclear to me how the candidate ancestors were selected, in particular whether they were selected after the filtering steps (in which case the ancestry analysis would be over-confident, since the simulations used individuals that cluster exceptionally well.)

On lines 965-999, we describe that the ancestor candidates were identified based on four steps: 1) they had their both parents' born within 80 km from each other, 2) they were removed by the spatial sampling procedure from the set of reference candidates, 3) they were arranged into geographic

groups based on their parents' birth places and 4) they were further filtered down based on PCA to have approximately similar genetic background among their group. We note that, in the selection process, no comparison between the ancestor candidates and the reference candidates were made using genetic data.

We agree that the ancestor candidates may be genetically less heterogeneous than an average individual with Finnish ancestry. However, we consider them suitable for our purpose of evaluating our pipeline with such real data for which we could reasonably confidently expect to know an "almost correct answer" to which we can compare the results. We have now added the following text to discussion lines 791-799:

"*The ancestor candidates that we used in our simulations were chosen by parental birthplace information and PCA of genetic data, and hence they are expected to be less genetically heterogeneous than an average individual with Finnish ancestry. Consequently, our results about the identifiability of an ancestor with certain genetic background are valid when the ancestor was approximately equally representative of their ancestry group as our ancestor candidates. We have not studied more complex scenarios, where an individual has a considerable proportion of genetic ancestry in a certain reference group, but that ancestry originates from many heterogeneous ancestors rather than one (or a few) homogeneous ancestor(s).*"

Figure 4 shows average ancestry over multiple simulated individuals. As I understand things, this would provide an estimate of the systematic bias in assignment, which is a relevant metric for the time-dependence analysis, but not of the uncertainty in assignment. I think this could be made clearer in the discussion of the results.

For all these reasons, the conclusions on lines 342-347 seem to overstate the accuracy of the regional inferences at the individual level. This is particularly important given that the manuscript highlighs forensics as a possible application of this type of research.

To show the variation in the ancestry estimates across the individuals, we include S10 Fig (previously Figure S7), S12 Fig and S14 Fig to the supporting information.

In addition, we made the following changes to the text:

- Line 354: *"(S10 Fig shows all the 20 individual ancestry profiles)"*
- Line 476: "*and S14 Fig for individual-level results for the major ancestry component.*"
- Line 529-539 (Previously lines 342-347): Revised the paragraph as follows:

  "The results averaged over individuals demonstrated that our reference groups are able to accurately detect ancestry all around Finland. With refset 2, we can identify Eastern and Western ancestry up to an accuracy of 6% (4 generations back). With refsets 6 and 10, the major source of ancestry is accurately detected 3 generations back in time and while the proportion of the minor ancestry is underestimated, the source of it can be identified 2 generations back. On the other hand, at the level of individual, the ancestry estimates show increasing variance with more heterogeneous genetic background (S10 Fig, S12 Fig and S14 Fig), which makes precise conclusions about genetic ancestry challenging for any one individual. Additionally, our ancestor candidates are likely to be less genetically mixed than an average individual with Finnish ancestry; hence our results do not necessarily directly apply to the Finnish individuals whose ancestors are more mixed."

- Line 801: Added a clarification (bolded):
  *"Our simulation results between A-East and A-West, **as well as between more detailed ancestor groups**, showed more variance in **the** ancestry estimates for individuals with more heterogenous background than for the homogenous individuals with ancestors from a single origin."*

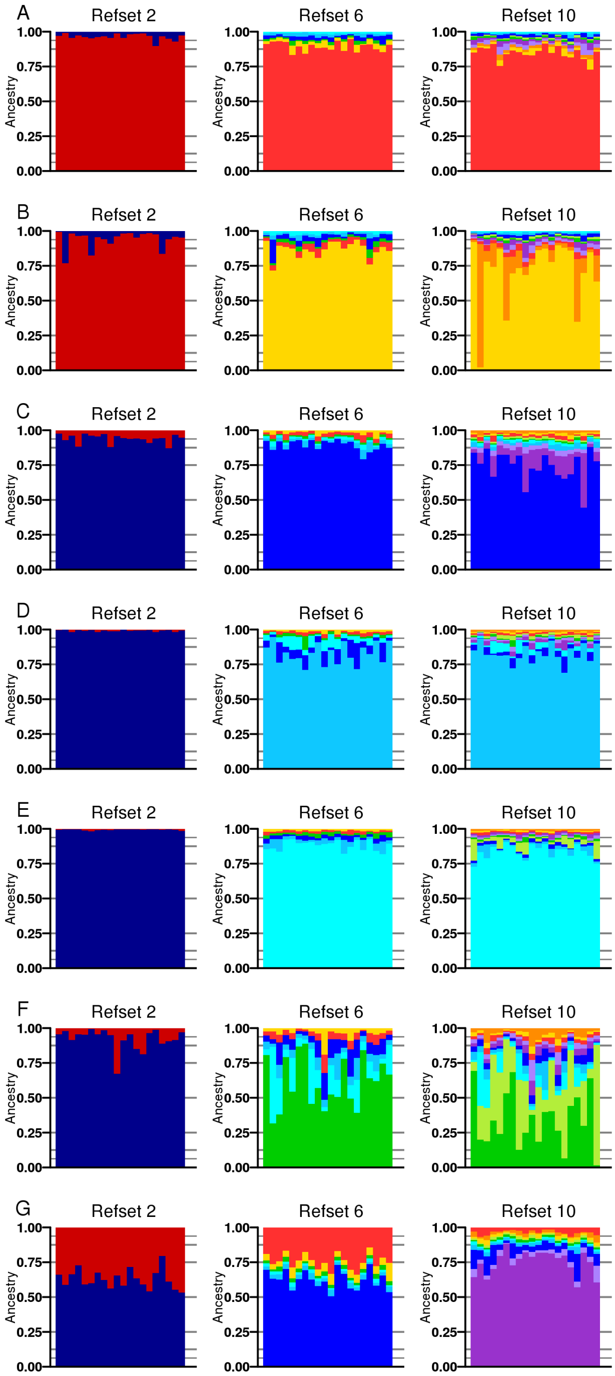

S12 Fig. Individual ancestry profiles for detailed single origin simulation scenarios.

The individual ancestry profiles for 20 individuals whose both parents originate from the ancestor candidate group of A) A-Southwest, B) A-Bothnia, C) A-N_Karelia, D) A-Kainuu, E) A-Kuusamo, F) A-Lapland and G) A-Evacuated (see Figure 4A for the mean values). The colors correspond to the reference groups in Figure 2.

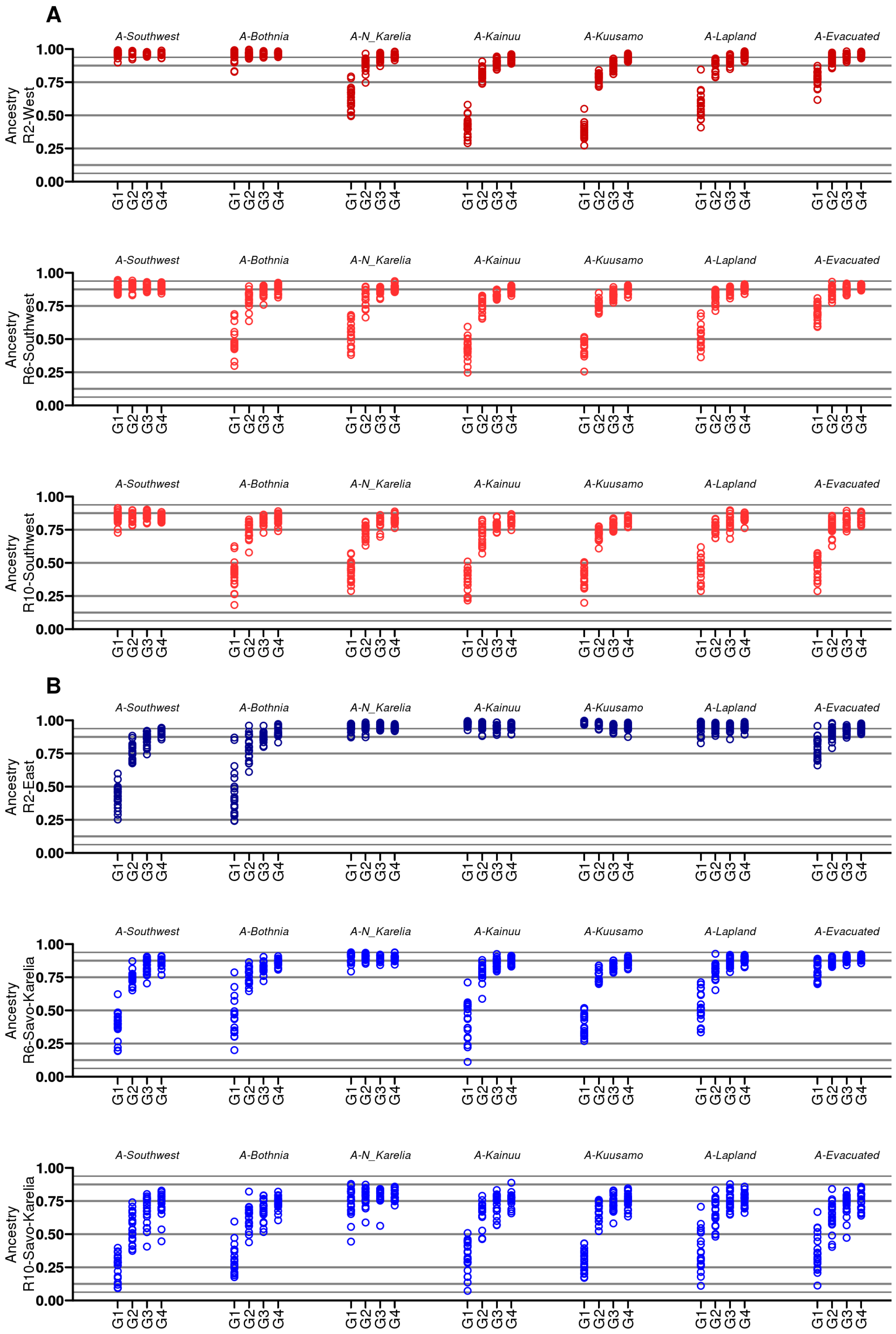

S14 Fig. Individual estimates of the major ancestry component in detailed simulation results.

Detailed simulation results for mixed ancestry from ancestor groups A-Southwest and A- N_Karelia (corresponding to S13 Fig). Panel A) presents individuals whose 2G-1, where G is the number of generations, ancestors originate from A-Southwest and 1 ancestor originates from the region in the title. Top row shows estimated ancestry in R2-West, middle row shows the same for R6-Southwest and bottom row shows them for R10-Southwest. Panel B) shows the same quantities for a simulation setting where all but one ancestor originate from A-N_Karelia and the reference groups whose estimates are shown are R2- East (top), R6-Savo-Karelia (middle) and R10-Savo-Karelia (bottom).

Minor points:

“homogeneous” and “distinct” reference groups – this has not quite been shown, and I would expect that a better powered or more detailed study would reveal that these groups are neither quite homogeneous nor quite distinct. Given that this work has a public outreach component, I would advocate for more careful language given how humans like to overinterpret genetic differences across groups. This is especially true here since the approach used extensive filtering to reach the “homogeneous” groups, and therefore the figures (such as Fig. 2) give an exaggerated idea of the divergence between populations.

This is an important point that we need to explain also on our website. We have used the term “reference group” rather than “population” to convey that we have carefully generated these groups by including only individuals that maximize the divergence between the groups, rather than, e.g., including the whole population living in a particular region of interest.

To avoid over interpretations, we have changed the wording “*homogeneous and distinct*” on line 194 and 751 into “*statistically separable*”.

Similarly, I would encourage the authors to avoid expressions such as “genetically intact”, which suggest a positive connotation to lack of mixing. (e.g., “genetically isolated” would be preferable).

As the topic of the manuscript might receive some public attention, we believe it is very important to be careful with the terminology. Thus, we have changed the word “*intact*” into “*little mixed*”. We have not used “genetically isolated” here, since this region of Ostrobothnia does not seem to be particularly strongly isolated compared to, for example, Kainuu (see new S23 Fig).

The sentence on line 56 now reads:

“*Additionally, different regions of Finland show very different levels of genetic mixing in 1900s, from little mixed regions like Ostrobothnia to highly mixed regions like Southwestern Finland*.”

We are also happy to discuss about any other sensitive wording that would need refining.

Reviewer1

(Line numbers refer to the marked-up copy of the manuscript.)

Reviewer's Responses to Questions

Comments to the Authors:
Please note here if the review is uploaded as an attachment.

Reviewer #1: This is an excellent paper that I thoroughly enjoyed reading. The methodology is sound and is in-line with the state-of-the-art in ancestry estimation. The results are very interesting, and the level of detail and precision on population movements within Finland are unprecedented. It is well-written, and well structured. I have only minor comments on the exposition to offer.

We thank you for reviewing our paper and for your positive and motivating words.

- My only criticism of this paper is that I found the terminology around the three "levels" of detail in which the population was studied very confusing. This made sense when discussed in the introduction, when talking about 2, 6 and 10 reference groups of ancestry. However, when you speak later of (say, line 188) about level-2 reference groups, and then around line 203 of level 10 and level 6, I thought you were talking about different heights in the FS-tree. It became clearer later on what you mean when I read the methods, but I think the terminology y could be a lot simpler and clearer here. Maybe use 2-way, 6-way and 10-way instead? This gives a better intuition to the reader that it's about splitting into groups, and "level" just seems misleading. I think "level" is being used in a few different ways, so it would be good to go through the text with this in mind.

Thank you for this comment. After considering possible terminology here, we ended up with an idea to define a term “refset”, as a shorthand for “reference set”, to refer to the three sets of reference groups that we are using. Thus, we have now rewritten the manuscript by replacing “level 2/6/10 reference groups” by “Refsets 2/6/10”. Conveniently, this term also shortens the text as we can avoid repeating the words “reference groups” in many instances. We introduce this term on page 8, line 174:

“*We refer to these three sets of reference groups with the term “refset” as an abbreviation for “reference set”.*

- Line 68: "A variety of methods" Now corrected.

- Line 75: The term "British Isles" is politically charged and regarded as offensive by many Irish people (please see the wikipedia page for a good summary: https://en.wikipedia.org/wiki/British_Isles_naming_dispute). Please consider avoiding this contentious terminology by using a more neutral term such as "Britain and Ireland".

Thank you for pointing out this contentious wording. We have changed the term “British Isles” to “*Britain and Ireland*”.

- Line 87. Delete "also". Now deleted.

- Line 91 (and elsewhere). Should it be "the Soviet Union"? Now corrected here and in the legend of Figure 1.

- Line 197: rerun -> reran. Now corrected.

- Line 337. This should be 2^{G - 1}, shouldn't it?

The value $2^G - 1$ means that when looking at, e.g., the ancestry at the level of 4 grandparents, i.e., 2 generations back in time (G = 2), then $3=2^2 - 1$ grandparents originated from A-Southwest and the remaining grandparent originated from the other ancestor group.

- Line 489. I couldn't parse this sentence - what is it trying to say?

We have rephrased the sentence (now on line 738):

"*With the ancestry profiles of the newborns, we cannot capture such local urbanization events that they have taken place mainly within a region of a single reference group."*

The sentence now reads:

*"**When such a local urbanization event happens within a region dominated by one of our genetic reference groups, it does not change the ancestry profile of the region, and consequently it does not show up in our results**."*

- Line 497. Swap "rather continuous" to "continuous rather". Now swapped.

Reviewer2

(Line numbers refer to the marked-up copy of the manuscript.)

Reviewer #2: In this paper, Kerminen and collaborators use state-of the-art population genetics tools to investigate the population structure of Finland, specifically to see how major events in the 20th century affected this structure. FineSTRUCTURE was used to partition a reference population into discrete subgroups, while SOURCEFIND was used to estimate the proportion of ancestry from each subgroup for each individual in a testing population, using entropy measurement and year of birth data to quantify the changes in ancestry over time.

The authors' findings matched what is known from the historical and demographic record, and further contributed that the migration of people from regions of Finland that were annexed by the USSR (and continue to be part of Russia to this day) have had the largest detectable effect on the population structure of Finland. Events such as the urbanization of the population in the latter half of the 20th century show much smaller, more local effects. Additionally, the study demonstrates the technical limits of SOURCEFIND to distinguish reliably ancestry proportions < ~5% from background population genetic variation. This has implications for inference of contributions further back in an individual's lineage.

Generally, I think the work done is of excellent quality and that the conclusions are supported by the results shown. I have three main comments on the methodology, and several minor suggestions:

We thank you for the careful reading of our manuscript.

Main comments:

1. Date of birth of "reference candidates".
Given the question asked, I would have thought that choosing the "reference candidates" based on earliest year of birth would have made sense, so that these reference groups really reflect the genetic background of the reference group early in the 20th century. I am not sure why the author decided not to choose reference individuals based on this info (l.514-518). In the >8000 potential candidates they reported, they made sure that they had good coverage across the country, but I feel that they could have consider selecting the people with the earliest date of birth as well. My concern is that, if a certain reference group is biased towards early ancestors (as reported l.516) and the other is biased towards later ancestors, this could potentially lead to strange effects when looking at admixture proportions in the regional subgroups. Similarly, were the ancestors in the simulations selected based on a logical date of birth scheme (ie. first ancestors are the oldest, with G1>G2>G3>G4)? If not, what is the expected impact of overlooking this aspect (that needs to happen in reality) on their results?

Thank you for asking these questions.

We agree that it would be logical to select the reference candidates to have the earliest birth years. However, in this work, our aim was to identify genetically motivated reference groups that allow fine-scale ancestry estimation throughout the country. Such a fine-scale estimation would have been considerably more limited if we had restricted our reference candidates to those born, for example, in years 1920-1929, as there were only 255 such reference candidates out of all 8,187 reference candidates.

The second concern is about the birth years of the reference groups and asks whether the age differences between the reference groups can affect the results. First, to clarify the age distributions of both the reference groups and the ancestor candidates, we have added S6 Fig and S4 Table (see below) describing the means and ranges of birth years to the supporting information. S6 Fig and S4 Table show that the range of mean birth years is similar across all reference groups and that the mean birth years of the groups vary within 1 year for refset 2, within 4 years for refset 6 and within 9 years for refset 10 (but only within 4 years if the group R10-Evacuated is not considered), which we think are small differences compared to the generation time. Statistically, it is only the group R10-Evacuated that shows a significantly reduced mean birth year (Mann-Whitney p-value 4.3e-7) but even this group included individuals born throughout the decades similarly to the other reference groups (S6 Fig). If the ages of reference groups were significantly different, a conceivable bias would be that the younger reference groups would falsely capture admixture patterns between the older groups. In our work, we

made efforts to control for such a scenario by excluding from the reference candidates the populations and individuals who showed signs of admixture as reflected by low identity proportions.

The last question asks whether the ancestors in simulations were selected based on logical birth order and what is an expected impact on simulations if they were not. We have added a schematic presentation of our simulation scheme (S7 Fig, see responses to minor comments) to our manuscript and it shows that we only sampled ancestors for the oldest generation of the simulation and then we simulated all the younger generations. The ancestor candidates, from which the ancestors for the oldest generation were sampled, were not controlled for their age and hence we may have simulated an offspring between individuals who were born at most 63 years apart. We expect that the age differences have little impact on the simulation results since even though the reference groups were defined purely by genetic similarity, they still ended up containing individuals throughout the range of possible birth years.

The following changes were made to the manuscript:

- Added S6 Fig and S4 Table to the supporting information.
- Line 263: Added a sentence "*The distributions of birth years for the reference groups are shown in S6 Fig and S4 Table*."
- Line 774: Corrected typo in p-value "3.4e-7" was changed to "4.3e-7".
- Lines 772: Updated (bolded) and added references to S6 Fig and S4 Table to the sentence:

  "*The mean birth year**, that was** 1953 **across all samples**, did not significantly vary between the reference groups, except for R10-Evacuated (mean birth year 1946, Mann-Whitney p-value **4.3e**-7) (**S6 Fig and S4 Table)**, whose region of origin was significantly affected by the Second World War.*"

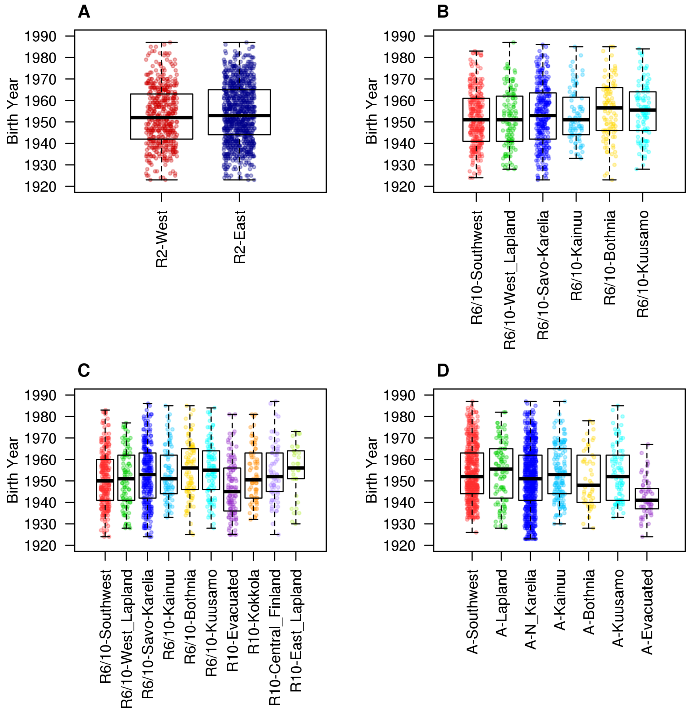

S6 Fig. Age distributions of reference groups and ancestor candidates.

Age distributions of the reference groups of A) refset 2, B) refset 6, C) refset 10 and D) the ancestor candidates. The boxplot whiskers show the range, the boxes show the interquartile range and the dark line shows the median of the birth years.

S4 Table. Range of the birth years (Min and Max) and the mean birth years of the reference groups.

Mann-Whitney p-value corresponds to a test between the focal group and the union of the rest of the groups at that refset.

	Min	Max	Mean	Mann-Whitney p-value
Refset 2				
R2-West	1923	1987	1953	0.09
R2-East	1923	1987	1954	0.09
Refset 6				
R6-Southwest	1924	1983	1952	0.05
R6-West_Lapland	1928	1987	1952	0.28
R6-Savo_Karelia	1923	1986	1953	0.68
R6-Kainuu	1933	1985	1953	0.68
K6-Bothnia	1923	1985	1956	0.04
R6-Kuusamo	1928	1984	1955	0.07
Refset 10				
R10-Southwest	1924	1983	1951	0.18
R10-West_Lapland	1928	1977	1952	0.62
R10-Savo_Karelia	1924	1986	1953	0.40
R10-Kainuu	1933	1985	1953	0.59
K10-Bothnia	1925	1985	1955	0.03
R10-Kuusamo	1928	1984	1955	0.02
R10-Evacuated	1925	1981	1946	4.3E-07
R10-Kokkola	1932	1981	1953	0.72
R10-Central_Finland	1925	1987	1954	0.49
R10-East_Lapland	1930	1973	1955	0.18
Ancestor candidates				
A-Southwest	1926	1987	1954	0.01
A-Lapland	1928	1982	1954	0.12
A-N_Karelia	1923	1987	1951	0.06
A-Kainuu	1930	1987	1954	0.18
A-Bothnia	1928	1978	1951	0.49
A-Kuusamo	1933	1985	1954	0.44
A-Evacuated	1924	1967	1942	1.7E-09

2. Admixture from outside Finland.
The authors mention in discussion the fact that the individuals could could have had ancestors from outside of Finland, and I am not really sure what the impact of that could be on the results presented, especially if that distribution of admixture is uneven between reference candidate or the tested regional subgroups. A solution would have been to "masked out" the chunks from distant ancestry in the genomes of individuals. And even leaving out recent immigration to Finland from countries all over the world, there has always been gene flow between Finland and Sweden (also probably Russia?). Could the authors show that these kind of admixture event would not (or only negligibly) bias their results?

Thank you, this is an important point to discuss.

In our paper, our starting assumption is that the genetic ancestry of the individuals is Finnish and this was checked by 1) the birth place information and 2) PCA within the study samples. We have now augmented these checks by performing another PCA together with the samples from the 1000 Genomes study, separately for each of the 5 continental super populations where the Finnish samples were excluded from the Europeans. By utilizing a K-nearest neighbors method, we identified 31 individuals who showed a closer affinity to the 1000 Genomes samples than to our reference group samples. We have now excluded these 31 individuals from our regional ancestry analyses. These exclusions did not noticeably affect the ancestry results or our conclusions.

We did these new exclusions because we think that the within-Finland ancestry profiles are not meaningful for individuals that rather cluster with the 1000 Genomes populations than with our reference samples. At the same time, we emphasize that we do not want to exclude any Finnish samples from the analysis simply because they may have a little bit more of ancestry from, say, Sweden or Russia, than an average sample as long as they still seem largely comparable to our reference groups so that the analysis remains meaningful. Given that there has always been migration between Finland and its geographic neighbors, it would not be easy to define which haplotype segments have already become also “Finnish “enough” rather than still being “Swedish” or “Russian”. For these reasons, we have removed only the strong outliers with a major part of their ancestry outside from Finland from these analyses, and we leave the connections between our reference groups and the neighboring populations outside of Finland as a topic for a future study.

We have made the following changes to the manuscript:

- Added a S16 Fig showing the excluded samples based on 1000 Genomes PCA to the supporting material.
- Line 879: Added the following sentences to describe the new PCA-based exclusions: “*Additionally, we ran PCA of our samples together with the non-Finnish samples from 1000 Genomes project[49]. By utilizing K-nearest neighbors method (K=21), we excluded 31 individuals who showed closer relatedness to the 1000 Genomes samples than to our Finnish reference candidates (S16 Fig).”*
- *Line 566: Updated the change in dominant ancestry in SOF between 1930 and 1980 from 24 into 23.*
- *Throughout the manuscript: Updated the number of samples from 18,494 to 18,463.*

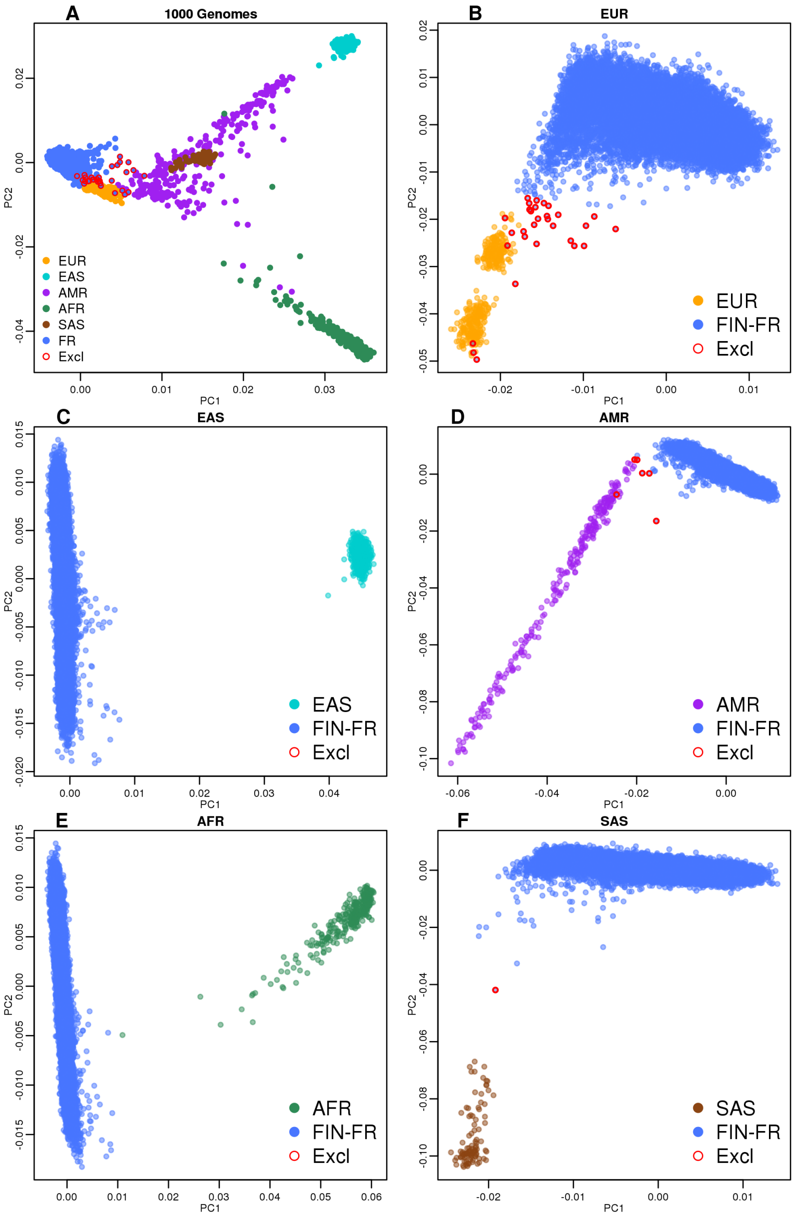

S16 Fig. Principal component analyses with 1000 Genomes samples and FINRISK samples.

A) PCA of 5 super populations of the 1000 Genomes (Phase 3) samples and our Finnish FINRISK (FIN-FR) samples. PCA of the FINRISK samples together with the B) non-Finnish European (EUR), C) East Asian (EAS), D) American (AMR), E) African (AFR) and F) South Asian (SAS) samples of the 1000 Genomes Phase 3. The FINRISK samples circled with red were identified to show admixture with one or more super populations and were excluded from the regional ancestry analyses. None of our reference individuals was among the excluded.

3. SOURCEFIND

I did not know much about SOURCEFIND before reading this manuscript and wish that there had been more justification for why it was used over, for instance, applying RFMix or other alternative methods? I would have liked to see more discussion of the implications of the fact that SOURCEFIND only seems to make accurate inferences about recent ancestry. How does this compare to other software? If someone were interested in making inferences about more remote ancestors, is there any existing software suitable to that question? How much of SOURCEFIND's uncertainty is a function of the specific population history of Finland? Would it be more or less accurate in a more heterogeneous population?

The first question was about why we chose FineSTRUCTURE for the estimation method. As we have previously utilized ChromoPainter and FineSTRUCTURE in our target population and shown that it performs well in fine-scale analyses (Kerminen et al. 2017) and SOURCEFIND is a state-of-art method built directly to work with these methods, we chose to build our ancestry estimation framework on it.

The next questions ask how SOURCEFIND compares to other methods and how much SOURCEFIND's uncertainty depends on the population history. While the scope our work was to focus on genetic ancestry within Finland, we know that SOURCEFIND has previously been compared to the classical ADMIXTURE (Alexander et al. 2009) analysis and to the previous version of ChromoPainter-based NNLS-method (Chacon-Duque 2019). In these comparisons, SOURCEFIND was shown to give highly concordant results with ADMIXTURE at continental population level and to outperform the NNLS-method in analyses with closely related populations. These results suggest that simpler methods, such as STRUCTURE, ADMIXTURE or PCA-based methods, are sufficient to capture genetic ancestry between remote populations, but computationally intensive haplotype-based methods are beneficial for more complex ancestry estimates. In addition, SOURCEFIND has been utilized to capture genetic ancestry at least within Europe (Saint Pierre et al. 2020, Byrne et al. 2020, Gilbert et al. 2019) and in Latin America (Chacon-Duque et al. 2019) demonstrating that the method gives sensible results also in other populations with different genetic backgrounds. Unfortunately, we are not aware of further comparisons between other methods, such as RFmix (Maples et al. 2013).

To summarize these aspects in the manuscript, we have added the following section to Discussion (lines 813-824):

"SOURCEFIND is a software tool that works directly on the output of the haplotype-based methods ChromoPainter and FineSTRUCTURE[9]. It has been previously utilized to capture genetic ancestry within Europe [12, 39, 40] and in Latin America[18]. We have previously shown that ChromoPainter and FineSTRUCTURE work well in our target population[27]. Therefore, SOURCEFIND was a natural candidate for testing how well our reference groups identify genetic ancestry. Previously, SOURCEFIND has been shown to give highly concordant continental-level ancestry estimates with a standard ADMIXTURE-analysis[8] and to outperform a ChromoPainter-based NNLS method [18]. We are not aware of a direct comparison between SOURCEFIND and other haplotype-based methods, such as RFmix[41]."

Minor suggestions:

- I had several questions on the simulation strategy while reading the results and I think that a figure, showing the simulation scheme graphically, would be beneficial to the reader. For example, it could clarify the fact that in the Almost-East/West simulations, the foreign ancestor was always drawn as a G1 ancestor (I think? from the results in Figure 3.. although I am not sure because legend says "a single ancestor, G generations back in time" l.271, suggesting it could be any G value?)

To clarify the simulation schemes, we have added a schematic representation of an example simulation to the supporting information (S7 Fig). We hope that this clarifies the fact that the ancestors were always sampled for the oldest generation G. For example, in a simulation scheme of Almost-East in G=2, one ancestor was drawn from West and the other 2^G-1 = 3 were drawn from East.

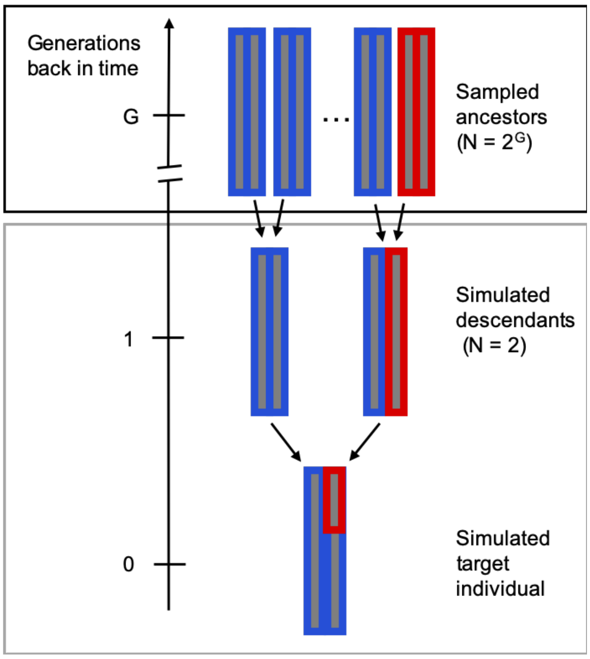

S7 Fig. Schematic representation of our simulation strategy.

In each simulation, 2^G individuals were sampled to represent the ancestors from G generations back in time (black box), where G varied between 1 and 5. All the subsequent descendants in generations G-1, G-2, …, were simulated to determine the genotypes of the target individual at generation 0 (grey box). In this example simulation, 1 ancestor is sampled from A-West (red) and the remaining 2^G – 1 ancestors were sampled from A-East (blue). The two adjacent bars correspond to the two haplotypes of an individual and the color corresponds to the ancestor candidate group.

The following changes were made to the manuscript:

- Lines 334-340: Added a paragraph:

  "We tested the identifiability of ancestry from different reference groups using simulations where 2^G individuals were sampled to represent the ancestors from G generations back in time (G varied between 1 and 5). We simulated the meioses within these ancestors, and within their subsequent descendants in generations G-1, G-2, …,1, to determine the genotypes of the target individual at generation 0. The ancestry of the target individual was then estimated and compared to the expected ancestry groups of the sampled ancestors based on their geographic and genetic origin (see S7 Fig for a schematic representation)."

- I am a bit confused with the wording "location of individuals" - does it refer to birth place, or where these individuals live/were sampled (eg. Figure 2)? Similarly, "parents' geographic location" and "parental birthplace information" are used... I think this wording should be classified throughout.

To elaborate this, we have made the following changes to the manuscript:

- Line 887: Added a part (bolded) to the sentence: *"To define geographically motivated reference groups, we first identified over 8,187 individuals whose parents were born within 80 km from each other* **and calculated their geographic location as the mean of their parents' birth places (available at the level of municipality).***"*
- Line 304: Included a sentence "*The locations were determined as the mean of the parents' municipalities of birth"* to the legend of Fig 2.
- Line 183: Rephrased (bolded) the sentence

  "*We used parental birthplace information to identify 2,741 geographically precisely located and evenly distributed unrelated individuals"*

  into

  "*We used* ***the municipalities of birth of parents*** *to identify 2,741 geographically precisely located and evenly distributed unrelated individuals"*

Similarly, I was a bit confused by the term "newborns" in several places (l.410,458,488) - what does this mean?

According to the Cambridge Dictionary (https://dictionary.cambridge.org/dictionary/english/newborn) the noun newborn refers to "a baby that was born recently". We use the word "newborn" in this meaning on lines 636 and 704 to describe the individuals who were born in the study regions but might have moved away at some point of their life.

- l. 647 "For individuals in the reference groups, the ancestry was estimated by leaving the individual itself out from the reference group." I am not really sure I understand how this is done in practice? Is FineSTRUCTURE rerun on the entire dataset by leaving one individual out?

ChromoPainter results were used as haplotype summaries where a reference candidate does not copy from him/herself when used as a donor individual in ChromoPainter analysis. Therefore, it was unnecessary to run FineSTRUCTURE again. Instead, we left the reference candidate out of the reference group in SOURCEFIND analysis when analyzing his/her ancestry proportions.

To clarify this, we have clarified the text and it now reads on line 962:

"For individuals in the reference groups, the ancestry was estimated by leaving the individual itself out from the reference group ***in the SOURCEFIND analysis****."*

- .432-433. absolute genetic diversity in regions is defined in opposition to average heterozygosity. I was curious as to whether average heterozygocity in the different regions has been computed as well, and if there are notable differences between regions or if it is quite homogeneous (maybe this has been done in a previous study, if so, please cite).

To our knowledge, average heterozygosity values have not been estimated for the study regions used here. To include information about the average heterozygosity in the manuscript, we have now evaluated PLINK's inbreeding coefficient F and included them in S23 Fig.

The following changes were made to the manuscript:

- Line 662: modified sentence

  "*Note that, with this measure, we do not attempt to quantify the absolute genetic diversity in each region, as measured, e.g., by the heterozygosity of individuals, but…."*

into

*"**With** this measure, we do not quantify the absolute **heterogeneity** in each region, as measured, e.g., by the average **inbreeding coefficient** of individuals **(S23 Fig)**, but…"*

- Line 1065: Added the description of how we estimated the ancestry:

  "Average inbreeding coefficient, F
  To complement the measure of change in heterogeneity of the ancestry profile per region, we also computed the average inbreeding coefficient, F, for each study region. The individual inbreeding coefficients were first calculated using PLINK 1.9 [46, 47] and then averaged over the individuals born in the region."

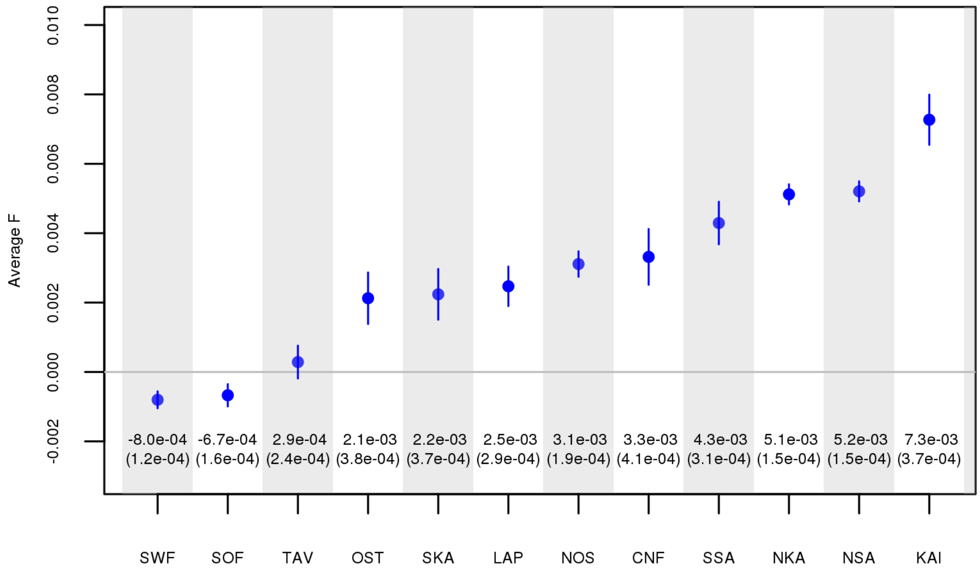

S23 Fig. Average inbreeding coefficient, F, per study region.

The values at the bottom of the figure report the average F per region with its standard error in parentheses. Whiskers show the 95% confidence interval.

- In my opinion, the term "admixture" is generally refering more to the mixing of genetic material from a distantly-related populations. I dont know what a better term would be though (gene flow? genetic mixing?)... or maybe it could simply be explicitely defined in the introduction that the terms admixed/admixture (normally used for more distantly related populations) will refer here to genetic mixing from/ gene flow between closely related populations.

Thank you. We have now tried to avoid the word admixture/admixed when talking about genetic mixing within Finland. We have made the following changes (bolded) to the manuscript:

- Line 235: "*suggesting that some populations were more admixed and/or so closely related to some other populations that they could not be reliably distinguished from the other populations*" →
  *"suggesting that some populations were more **mixed** and/or so closely related to some other populations that they could not be reliably distinguished from the other populations".*

- Line 370: "*For the individuals with some admixed background (Almost-West, Almost-East)*" →
  "*For the individuals with **mixed** background (Almost-West, Almost-East),"*

- Line 375: “*While this may well reflect a small but real admixture proportion in our ancestor candidates*” →
  “*While this may well reflect a small but real **ancestry** proportion in our ancestor candidates*”

- Line 506: “*This suggests that we may overestimate the R10-Evacuated component for an individual who happens to be admixed between eastern and western Finland, possibly because the R10-Evacuated group itself shows some admixture between R2-East and R2-West (Fig 4A A-Evacuated)”* →
  *“This suggests that we may overestimate the R10-Evacuated component for an individual who happens to be **mixed** between eastern and western Finland, possibly because the R10-Evacuated group itself shows some **mixing** between R2-East and R2-West (Fig 4A A-Evacuated).”*

- Line 510: “*Other pairs of regions do not show similar admixture effects.”* →
  *"Other pairs of regions do not show similar **results of mixing (S15 Fig).”**.*

- Line 743: “*While our genetic analysis results match well with the known history, the exact interpretation of ancestry and admixture is complicated because it always depends on the available reference groups*” →
  “*While our genetic analysis results match well with the known history, the exact interpretation of ancestry and **genetic mixing** is complicated because it always depends on the available reference groups*”

- Line 950: “*SOURCEFIND uses an averaged chromosome painting of the reference groups to find the admixture proportions for the test individual/population utilizing an MCMC method.*” →
  “*SOURCEFIND uses an averaged chromosome painting of the reference groups to find the **ancestry** proportions for the test individual/population utilizing an MCMC method.*”

Website:

The website is great and very appealing! However, on Safari I see a truncated version (the right side goes outside the page and we can't slide the page - at least on two different computers). Also, on iPhone, the ancestry/tree panel is not displayed at all (might be too heavy for mobile - but just wanted to report it).

Thank you for letting us know. We have asked our web team to work on these issues.

Typos

- l.140-142 "We will first introduce a procedure to identify suitable reference groups, then we test their performance to detect ancestry via simulations, and finally apply them to estimate the ancestry of 18,494 FINRISK samples to characterize" >> I am not sure what "their performance", "them" refers too, probably to the methods?

We have rephrased the sentence on line 164 from

“*… then we test their performance to detect ancestry via simulations”*

into

“… then we use simulations to test the performance of our reference groups in detecting ancestry”.

- l.588 "For birth region analyses, we further excluded individuals marked to had been born in the municipality of Karjala" >> ... to have been born ...

We have corrected this.

- l.708 "We estimated the rate of change for whole time period from 1923 to 1987" >> ... for the whole time period ...

Now corrected.

- Some figures are missing labels on axes (eg. Figures 3,4, S7-10)

We have added the missing labels to Figures 3, 4, S7, S8, S9 and S10.

Other changes in the manuscript

(Line numbers refer to the marked-up copy of the manuscript.)

- Included section Supporting information captions listing the captions of supporting figures and tables.
- Updated Figure, Table and sample numbers.
- Generated tittles and updated legends for the figures.
- Line 868: Corrected the heterozygosity threshold used.
- Line 1124: Added the URL source of map borders.
- Line 913: Specified that PCA was performed on ChromoPainter's coancestry matrix
  "… and 3 PCA outliers, resulting in 2,741 individuals." →
  "… and 3 outliers ***in PCA on ChromoPainter's coancestry matrix as described in*** *[9], resulting in 2,741 individuals.*"
- Line 997: Added a specification (bolded): "***We did not use the genetic data of our reference individuals or*** *SOURCEFIND estimates were not used in the selection process of the ancestor candidates."*
- Lines 1000-1015: Added a section *Principal component analyses* to describe PCA and moved part of the "Selection of ancestor candidates" there. The section reads:

  *"**Principal component analyses***
  We performed principal component analyses both within the FINRISK data and together with the samples of the 1000 Genomes Project[49] using PLINK 1.9 [46, 47].

  Principal component analysis within FINRISK samples was performed for 18,719 individuals and 56,661 LD-independent variants. These data included 256 individuals who were not part of the haplotype-based analyses as they were only later excluded due to ambiguous or missing location data or as outliers of the 1000 Genomes PCA (S16 Fig). LD-independent variants (56,661) were defined using command --indep-pairwise with 1500 kb window size, 500 kb step size and 0.2 as r^2 threshold in PLINK 1.9, and by further excluding the long-range LD regions described in [51].

  Principal component analyses together with the 1000 Genomes data were performed on 18,715 FINRISK samples and 1,536 non-Finnish samples of the 1000 Genomes phase 3 data using 49,423 LD-independent variants. We performed 6 separate PCA runs: one with all five super populations (314 Africans, 264 Americans, 480 East Asians, 380 Europeans, and 98 South Asians) *together with the FINIRSK samples, and also 5 runs, where each super population was separately analyzed with the FINRISK samples (S16 Fig)."*

- Corrected typos and wording as shown in the marked-up copy of the manuscript.
