# Supplementary material for: Changes in the fine-scale genetic structure of Finland through the 20th century
Source: PLoS Genet. 2021 Mar 4;17(3):e1009347. doi: 10.1371/journal.pgen.1009347 (PMC7932171; doi:10.1371/journal.pgen.1009347)
Supplement: Attachment — Submitted filename: Responses_PlosGen_review_round2_v1.pdf [file pgen.1009347.s031.pdf]

Responses to the reviewers' comments

Reviewer #1:

The authors have addressed all of my points well, I have no further comments.

We thank reviewer 1 for taking time to review the manuscript.

Reviewer #2:

(Page and line numbers refer to the marked-up copy of the manuscript.)

I thank the authors for answering my questions and addressing them in the manuscript. I think the changes they made have greatly improved the readability of the paper and have made it more comprehensible.
I have two final minor comments:
- Line 448: "whence" means "from which" or "from where". I think the authors mean "where" or "for which" here.
- On the issue of the word "newborns" : When this term is used, it's usually referring to people who are currently newborns or to discuss some facet of infancy where being newborn is relevant (for example, "newborns can't focus their eyes"). It's not generally used the way the authors have used it in this paper. I would use the clunkier, but more accurate "individuals born in 19XX" in its place.

Thank you for your comments. We have now corrected these:

- Line 456: *"whence"* → *"with"*
- Line 434: *"newborns"* → *"individuals born around 1950"*
- Line 485 (Fig 7 caption): *"The rate of change is calculated by regressing the entropy of the region-specific ancestry profiles of the* ***newborns*** *with respect to refsets 2, 6, and 10 on the year of birth."*
  → "The rate of change is calculated by regressing the entropy of ancestry profiles of the individuals born in the region on their year of birth. Entropy was calculated with respect to refsets 2, 6, and 10."

Reviewer #3:

(Page and line numbers refer to the marked-up copy of the manuscript.)

This paper by Kerminen and al. is simultaneously addressing the question of genetic structure of Finland, which is not totally new and the properties of SOURCEFIND algorithm when the source populations show small differentiation (at the fine greographical scale, within one country, be it Finland).
Revisiting the genetic structure of Finland and brings a very interesting approach, the analysis of rapid change in time of this structure, because of dramatic events. In this regard, the approach is original and worth reporting.

We thank the reviewer for the comments and are happy that the approach is considered to be original and worth reporting.

The introduction/title could provide more clear description of the study's goal. This work basically tests, using simulation from realistic data, whether SOURCEFIND can correctly identify origin when the source population display only limited difference. Moreover, in my opinion, this introduction should stress earlier one of the original points, the possibility to stratify the changes in genetic composition within a short period of time.

Thank you for the suggestions. We consider that the primary goal of the study is to report "Changes in the fine-scale genetic structure of Finland through the 20th century". Hence, we have left our title as it is. This primary goal is made clear as early as in the 3rd sentence of Abstract and therefore we have chosen to leave the ordering of paragraphs in Introduction as it is, proceeding from the existing work to the new components of this study.

I don't find very clear the references of the use of Globetrotter and Sourcefind. The message seems to be that the methods have been applied to populations that are large whereas in this study it is going to focus on fine-scale structure of a supposedly less broad populations. However, the referenced studies focus for instance on Ireland. I guess here it would be more clear to explictely state that you are testing these methods in a fine-scale context where source populations and target populations are very close. And where source populations are not heterogenous.

Thank you. We agree that it is important to communicate more clearly that we test the ancestry estimation using fine-scale source populations within a single country. Therefore, we have rephrased this part in Introduction and it now reads on page 4, lines 76-82 (changes in bold):

*"So far, the haplotype-based methods, such as GLOBETROTTER[17] and SOURCEFIND[18], have been applied to estimate ancestry and date admixture **from** relatively broad geographic areas, for example, **in** Europe[11, 13, 19, 20], Africa[21-23] and Eurasia[24, 25]. Consequently, there remains limited information about the accuracy and robustness of individual-level ancestry estimation **using** fine-scale **source populations**. In this study, we assess these questions within a single European country, Finland."*

In terms of novelty the description of the genetic structure of Finland in present times has already been adressed and I find that they don't separate enough, even in the second part, what is really new from what is not. I think that the contribution of refugees from Soviet Karelia is new. Also, the evolution of the genetic structure in a short period of time is something important - actually, this is in my opinion the most important point. This is also quite new compared to previous papers where we could only estimate the change in population size from current genomes in the different clusters.

We agree that genetic structure of Finland is not new, although it is presented with more details here than in previous publications. We make clear that we replicate the previous results when we first time discuss our results about population structure in Finland on page 9, line 163.

*“In **Error! Reference source not found.**, we confirm that our results closely match with previous results on the fine-scale structure in Finland[27, 32]…”*

The reviewer is right that both the genetic traces of WWII and the annual evolution of genetic structure are new components in this study. We make this novelty clear in Introduction page 6, line 120: *“To our knowledge, this is the first study to track geographically the annual genetic contributions of subpopulations within a single European country.“*

Concerning the simulations, I find it very interesting to start from existing chromosomes representative of a region in order to see Globetrotter's ability to estimate the proportions of origin of each population. They start from existing chromosomes (estimated in any case) as "founders" and simulate transmission. Somewhat in the spirit of HapGen which was distributed with the 1000 genomes. The "critics" asking to take founders with an older date of birth miss the fact that the problem is just theoretical: let's take ancestors representing populations with an Fst close to the classical Fsts between provinces.

The simulations seem to me to be valid in relation to the question posed. One thing has not been accounted for, however. This is the fact that they only capture a fraction of the haplotype diversity because they take the chromosomes as they are and observed whereas one could imagine generating generating founder haplotypes using the observed “source population” haplotypes but allowing for recombination at this stage – in the ancestors. Thus, they could have captured a wider and more accurate haplotype diversity while still relying on the observed structure. This is however a limited criticism as it is still of matter of simulating given a Fst and testing the consequences in lower generations. The simulation process (including the algorithm to identify “seed” founder populations) is more clear in this reviewed versions, as asked by editor and reviewers in the first round.

Thank you for the detailed thoughts about the simulation process. You are correct that we use the observed haplotypes for the founders in our simulations. We note that when we simulate genetic data through up to 5 generations, at each non-founder generation, we do make new haplotypes from the population distribution according to the recombination process.

This paper (which seems to me very good and very pro) seems to chase two hares at the same time ... this is what is a bit annoying because it mixes a practical problem (structure and history of Finland) and a theoretical one - which uses very realistic (because real) data and therefore in a context - structure Finland.

Thank you for the comment. We consider the results about changes in fine-scale genetic structure as the primary goal of this study and the simulations as a supporting analysis to validate our methodology and to be able to interpret our primary results. We agree that such a simulation study could already be of interest on its own.

The problem is interesting because it seems to me that Globetrotter seems to have been made to find admixture (and date it) from much more differentiated source

populations. So it is and see the properties of the method to the extreme.
Even if this paper is a bit confusing because it piles up two (nearly three) topics, it has this novelty of following genetic structures on several generations and therefore comparing the impacts of internal migration (in the sense of the same people) and urbanization. Results from simulations can also give useful guidelines for interpreting SOURCEFIND results from other populations.

Thank you for your comments.
